# A Genome-Wide Approach to Discovery of Small RNAs Involved in Regulation of Virulence in *Vibrio cholerae*

**DOI:** 10.1371/journal.ppat.1002126

**Published:** 2011-07-14

**Authors:** Evan S. Bradley, Kip Bodi, Ayman M. Ismail, Andrew Camilli

**Affiliations:** Howard Hughes Medical Institute and the Department of Molecular Biology and Microbiology, Tufts University School of Medicine, Boston, Massachusetts, United States of America; Yale University, United States of America

## Abstract

Small RNAs (sRNAs) are becoming increasingly recognized as important regulators in bacteria. To investigate the contribution of sRNA mediated regulation to virulence in *Vibrio cholerae*, we performed high throughput sequencing of cDNA generated from sRNA transcripts isolated from a strain ectopically expressing ToxT, the major transcriptional regulator within the virulence gene regulon. We compared this data set with ToxT binding sites determined by pulldown and deep sequencing to identify sRNA promoters directly controlled by ToxT. Analysis of the resulting transcripts with ToxT binding sites *in cis* revealed two sRNAs within the *Vibrio* Pathogenicity Island. When deletions of these sRNAs were made and the resulting strains were competed against the parental strain in the infant mouse model of *V. cholerae* colonization, one, TarB, displayed a variable colonization phenotype dependent on its physiological state at the time of inoculation. We identified a target of TarB as the mRNA for the secreted colonization factor, TcpF. We verified negative regulation of TcpF expression by TarB and, using point mutations that disrupted interaction between TarB and *tpcF* mRNA, showed that loss of this negative regulation was primarily responsible for the colonization phenotype observed in the TarB deletion mutant.

## Author Summary

*Vibrio cholerae* is the causative agent of the diarrheal disease cholera, which remains a significant public health issue in Africa, South Asia and recently Haiti. To better understand virulence gene regulation in *V. cholerae* we sought to investigate the contribution of small non-coding regulatory RNAs (sRNAs) to regulation of virulence in *V. cholerae*. We undertook a genome wide approach to sRNA discovery combining direct sequencing of sRNA transcript cDNA and genome wide binding studies of the master protein regulator of virulence, ToxT. This approach yielded one previously known and 17 new potential sRNAs under the control of ToxT. We investigated one of these new sRNAs and showed that it negatively regulates expression of the secreted colonization factor TcpF, adding a new facet to the complex gene regulatory network necessary for virulence in *V. cholerae*.

## Introduction

*Vibrio cholerae* is the causative agent of cholera [1], a disease characterized by voluminous secretory diarrhea that is frequently fatal in the absence of treatment [2]. Cholera is endemic in parts of South Asia and Africa and is capable of causing massive epidemics whenever clean drinking water is lacking. While the precise *in vivo* signals that lead to expression of the pathogenesis program in *V. cholerae* have not yet been determined, the regulatory events leading to expression of the primary virulence factors, cholera toxin (CTX) and the toxin co-regulated pilus (TCP), have been well studied and the major protein factors in the cascade have been identified [3], [4]. Central to transcription of the major virulence factors is production of the AraC family transcriptional activator ToxT [5], [6]. ToxT activates production of CTX and TCP by binding to sequences known as *toxboxes* upstream of the −10 and −35 promoter elements in those operons and stimulating transcription [7], [8]. ToxT has also been shown to inhibit expression of the mannose-sensitive hemagglutinin (MSH) pilus, which is an anti-colonization factor, both by stimulating its degradation and inhibiting its transcription [9]. Expression of these and other factors during infection is dynamic [10]–[12] presumably due to rapidly changing conditions within the small intestine as the infection proceeds. We hypothesized that some steps in this dynamic expression may be controlled by ToxT-regulated small non-coding RNAs (sRNAs). Such regulators would have the advantage of being fast acting since an sRNA need only be transcribed in order to function.

sRNAs influence a variety of processes in bacteria, mostly at the post-transcriptional level through sRNA-mRNA interactions [13], [14]. Processes impacted by sRNA regulators include the DNA damage (SOS) response [15], [16], sugar uptake [16], quorum sensing [17], [18], expression of outer membrane proteins [19], [20] and many others. Recent investigation into the sRNA transcriptome of bacteria has indicated much greater complexity than was previously appreciated [16], [21]–[23]. Given that sRNAs are such ubiquitous regulators of gene expression, we were interested in investigating whether they contributed to virulence factor regulation in *V. cholerae*.

There are several pieces of evidence that suggest the existence of sRNA regulators of virulence in *V. cholerae*. The major sRNA chaperone Hfq, a protein which many sRNAs act in conjunction with, is required for *V. cholerae* pathogenesis [24]. In addition, two sRNAs that contribute to virulence were recently discovered. The first regulates the porin OmpA and outer membrane vesicle formation [20] but is not under the control of the virulence regulon, while the second regulates glucose uptake and is a member of the ToxR regulon as it is transcriptionally activated by ToxT downstream of ToxR [25]. To conduct a thorough survey of the possible ToxT-regulated sRNAs, we took a genome-wide approach to discover sRNAs involved in virulence gene regulation by direct cloning and sequencing of sRNA transcripts and by identifying genomic sites bound by purified ToxT.

## Results

### Detection of putative ToxT-regulated sRNA transcripts

We used direct cloning and deep sequencing of RNA transcripts 50–250 nucleotides in length [16] to compare a culture in which ToxT or an inactive version missing the helix-loop-helix DNA binding domain (ΔHLH) [11] was expressed from an arabinose inducible promoter on a plasmid (pToxT or pToxTΔHLH). The highly abundant 5S rRNA and tRNAs present in this size range were depleted prior to sequencing as described [15]. After sequencing we removed residual tRNA and rRNA reads and aligned the remaining reads to the *V. cholerae* genome. The number of reads of each unique transcript in each library was normalized to the number of reads of MtlS, an abundant sRNA [16] that does not vary between the conditions tested here (data not shown). A total of 14,578 unique sequences were identified between the two libraries, of which 13,309 were present in only one library or the other. Many sequences not shared between the libraries were very low in abundance and may represent products of random RNA degradation either *in vivo* or during preparation of the libraries. The positions of all reads aligned to the N16916 genome and their relative abundances in the two libraries is shown in (Table S5). The short sequencing reads were organized into clusters to provide an approximation of each putative sRNA sequence. Many of the 1,269 clusters shared between the libraries had large variations in abundance between the libraries. While this may reflect the true difference in the sRNA transcriptome between these two strains, to help us narrow the list of potential sRNAs we sought a method to determine which sRNAs were directly regulated by ToxT.

**Figure 1:**
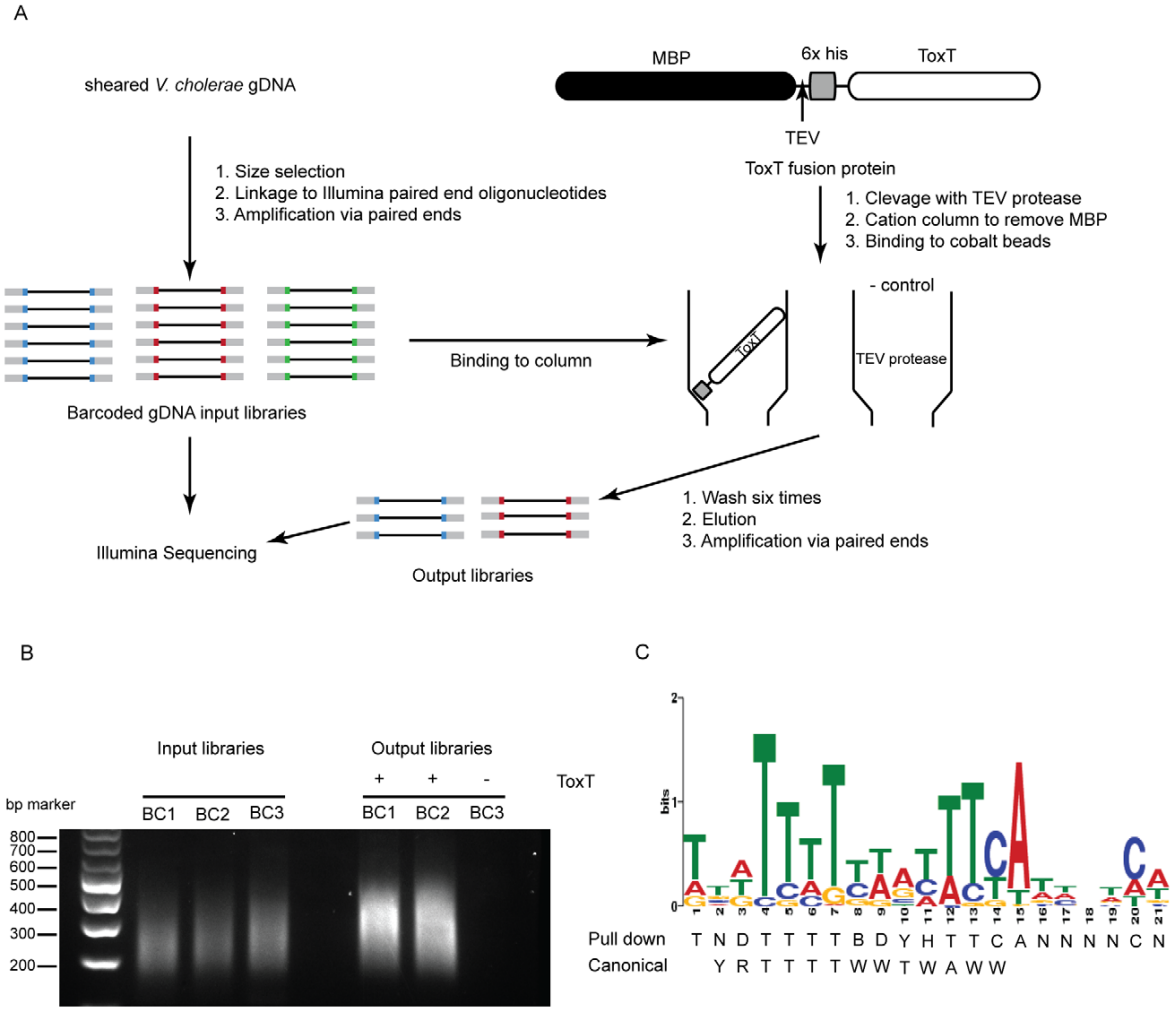
Affinity purification of ToxT binding sequences. **A**) Experimental outline of the ToxT *in vitro* DNA pulldown. Because the purification procedure left a residual amount of TEV protease in the His-ToxT prep, a negative control pulldown was performed with 6His-TEV protease. **B**) Amplification of resulting libraries after pulldowns shows that in the presence of ToxT (libraries BC1 and BC2), DNA was eluted from the column, whereas with TEV bound to the column instead, no DNA is detected after 10 cycles of amplification (BC3). **C**) The resulting binding motif predicted for ToxT present in 66 out of 67 pulldown sites with an E-value of 2.3e14 as analyzed by MEME software according to parameters detailed in Methods. The ToxT binding motif predicted by pulldown is shown with the previously reported canonical *toxbox*. Single letter codes are as follows; B = C/G/T, D = A/G/T, H = A/C/T, N = A/C/G/T, R = A/G, W = A/T, Y = C/T.

Because sRNA promoters share many characteristics with open reading frame promoters, it seemed reasonable that any sRNA directly controlled by ToxT would have a ToxT binding site in *cis*. To investigate this we undertook a genome-wide ToxT pulldown of genomic DNA fragments 200–500 bp in length that were modified to allow for subsequent deep sequencing (figure 1A and 1B), similar to an approach taken with the transcription factor CodY from *Staphylococcus aureus* [26]. Using a cut off of 3-fold enrichment in pulldown libraries over input libraries, we identified 199 putative binding sites of which 67 overlapped between technical replicates and likely represented the most specific sites (table s6). A DNA binding motif generated from the 67 enriched sites was a close, though not identical, match to the canonical *toxbox* [27] (figure 1 panel C). Of the overall 199 putative binding sites, 64 mapped to the *Vibrio* Pathogenicity Island (VPI), which is consistent with the fact that this locus contains the majority of ToxT-regulated genes. Most, but not all previously described ToxT binding sites were present in the pulldown library, notably absent are sites within the *tcpA* promoter [27] and sites within the MSH pilus operon [9].

**Figure 2:**
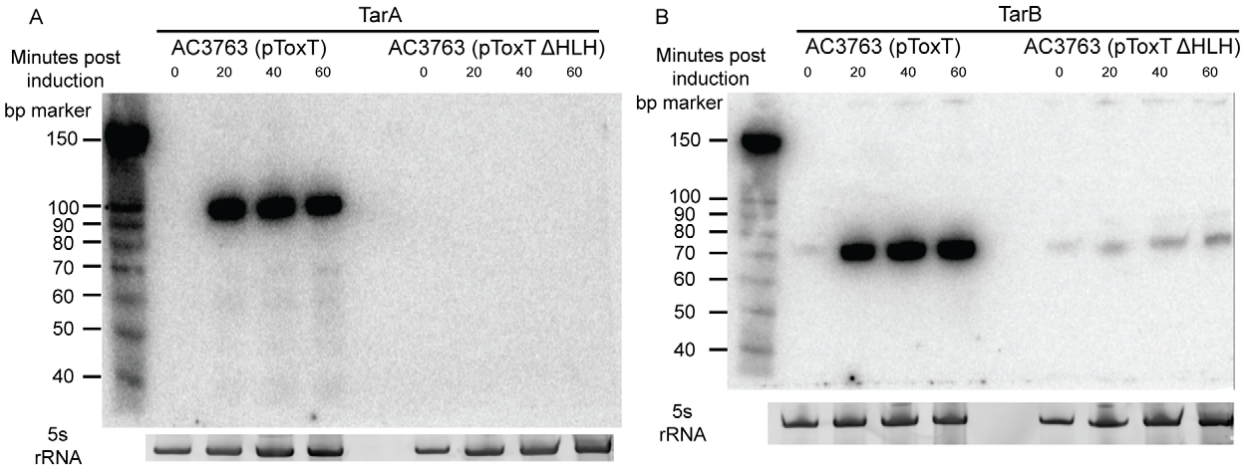
Northern blots of TarA and TarB. ^32^P-UTP labeled riboprobes complementary to sRNAs were used to blot for the presence of the expected sRNAs in total RNA isolated from cultures expressing ToxT or ToxTΔHLH from plasmids. A) TarA is detected at the predicted molecular weight and is present at high abundance within 20 minutes after induction by addition of arabinose, which is absent in the transcriptionally inactive ΔHLH form of ToxT. B) TarB is also present at the predicted size based on sequencing data and also shows dramatic upregulation in the ToxT expressing strain but not the strain expressing inactive ΔHLH ToxT.

Cross-referencing the putative ToxT binding sites with ToxT-regulated sRNA sequencing data yielded a collection of 18 potential sRNAs transcribed from intergenic regions with *cis* ToxT binding sites. The locations of these pulldown sites, sRNA transcripts and relative abundance between ToxT and ToxTΔHLH expressing strain libraries are shown in table 1. This analysis revealed two putative sRNAs within intergenic regions in the VPI. To investigate whether these two sRNAs represented genuine transcripts, we probed for each by northern blot using total RNA from cultures expressing ToxT or ToxTΔHLH. Both of these sRNAs are dramatically upregulated upon expression of ToxT and both are present at the expected size predicted by the sRNA deep sequencing experiment (figure 2). One of these sRNAs was discovered independently by another group and was named TarA [25] (for ToxT activated RNA A). The other, to the best of our knowledge, remains uncharacterized. Since it also showed dramatic up regulation upon expression of ToxT, and given its role in virulence (described below), we named it TarB. Having now determined that at least two ToxT-regulated sRNAs were present in the VPI, we set out to determine whether they played detectable roles in the virulence of *V. cholerae*.

**Table 1:** Intergenic sRNAs with *cis* located ToxT binding sites.

Enriched pulldown genome coordinates		sRNA sequencing read genome coordinates^a^		nearby ORFs	ORF annotation; sRNA annotation	Normalized ToxTΔHLH library reads^a^	Normalized ToxT library reads^a^
Start	End	Start	End				
134659	134803	134505	134394	VC0142/VC0143	hypothetical/hypothetical	155	375
149092	149248	149280	149445	VC0157/VC0158	alkaline serine protease/glutamate racemase	0	413
177452	177653	177267	177165	VC0176/VC0177	transcriptional regulator (putative)/hypothetical	242	5630
523047	523177	522904	522819	VC0489/VC0490	hemolysin (putative)/conserved hypothetical	786	167
889129	889314	888622	888550	VC0825/VC0826	*tcpI*/*tcpP*; ***tarA***	0	2182
911227	911352	911310	911233	VC0845/VC0846	putative lipoprotein/phage integrase (degenerative); ***tarB***	2922	519
1037594	1037784	1037758	1037862	VC0971/VC0972	*ligA* DNA ligase/porin, putative	851	605
1198742	1198847	1199141	1199239	VC1130/VC1131	*vicH* DNA binding protein/membrane binding protein (putative)	798	3
1412924	1413078	1413070	1413198	VC1328/VC1329	*D*-galactose or *D*-glucose ABC transporter, permease protein/hypothetical	0	257
2168242	2168432	2168188	2168097	VC2013/VC2014	PTS system, glucose-specific IIBC component/conserved hypothetical	1078	143
2433764	2433903	2433569	2433387	VC2278/VC2279	membrane protein, putative/*pepD*	38	10
2549682	2549799	2549443	2549565	VC2384/VC2385	conserved hypothetical/DNA polymerase	3481	2463
2552739	2553002	2553119	2553020	VC2387/VC2388	conserved hypothetical/hypothetical	0	71
180027	180205	182477	182600	VCA0161/VCA0161	tryptophanase *tnaA*/tryptophan leader peptide *tnaC*	0	198
214601	214731	214400	214531	VCA0197/VCA0198	GMP reductase (*guaC*)/DNA methyl transferase (putative)	264	205
485328	485491	485507	485625	VCA0546/VCA0547	conserved hypothetical/hypothetical	0	525
885950	886158	885901	886039	VCA0932/VCA0933	hypothetical/cold shock domain family protein	0	3654
886713	886820	893566	893461	VCA0934/VCA0935	hypothetical/hypothetical	1134	448

a Overlapping clusters hypothesized to represent the same transcript were pooled to determine putative starts and stops and normalized abundances. Normalized abundance scores were rounded to the nearest whole number.

### Assessment of sRNA mutants in the infant mouse model of intestinal colonization

**Figure 3:**
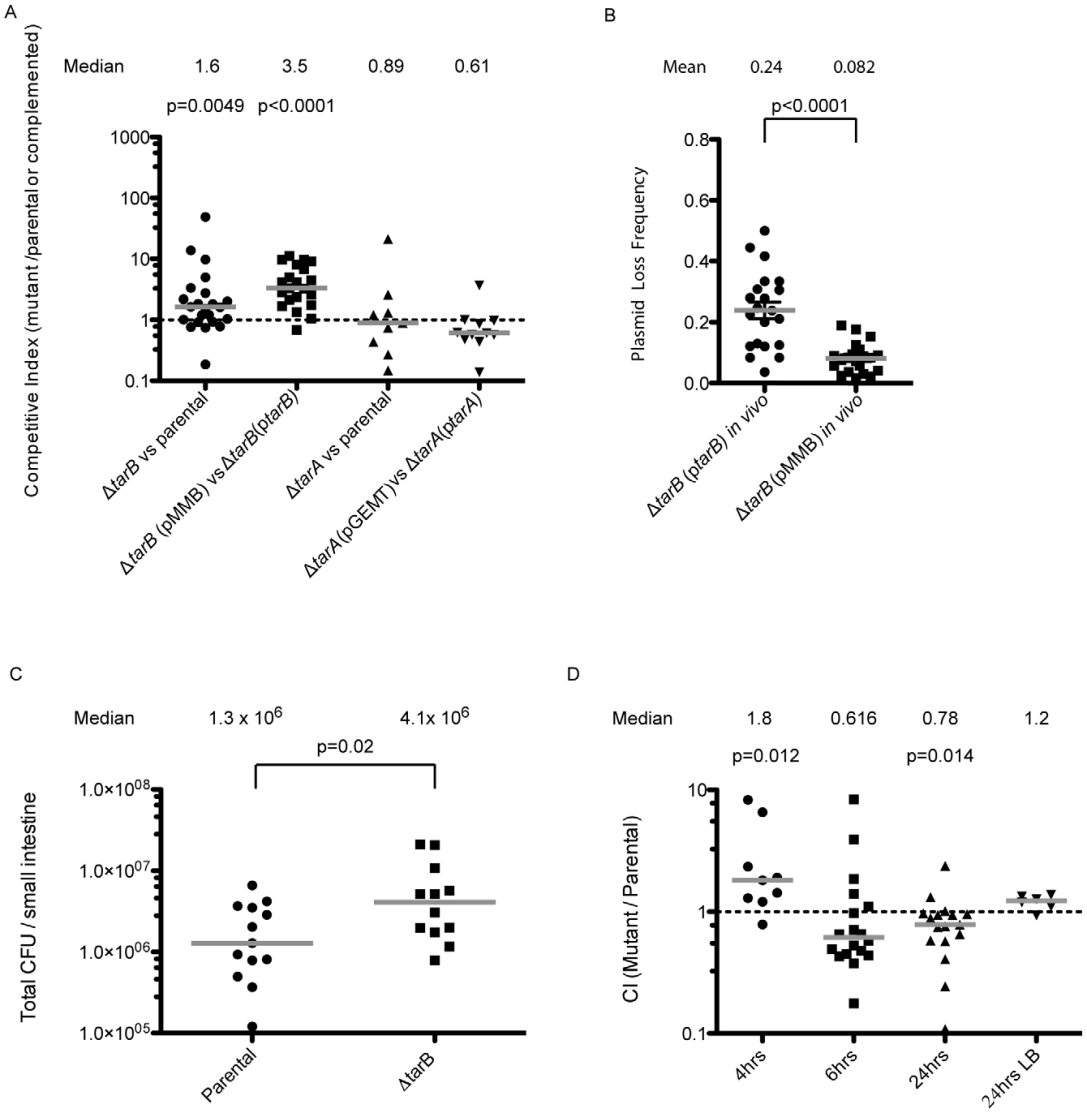
Mouse infections performed with ΔsRNA and complemented strains. **A**) Competitions performed with unmarked deletions of *tarA* and *tarB* against the parental strain carrying a *lacZ* deletion. Competitive indices are reported as the ratio of CFUs in the output adjusted for the input ratio. The Δ*tarB* strain shows enhancement of colonization over the parental strain, but the Δ*tarA* strain shows no significant trend. When *tarA* and *tarB* (promoters included) were cloned into complementation vectors and the complemented strains were competed against deletion strains carrying vector alone, the Δ*tarB* strain shows a more dramatic enhancement of colonization over the complemented strain (median CI = 3.5), while the Δ*tarA* complemented strain shows a slight but not significant advantage over the deletion strain (median CI = 0.61; Wilcoxon signed rank test on log transformed data). **B**) Output plates from the competitions in panel A were replica plated onto plates containing ampicillin to assay for presence of the plasmid. Replica plating shows that p*tarB* is lost 2.9× more frequently then the vector alone (p<0.01, two sample T-test with Welch's correction for unequal variance). C) Single strain infections were performed with wildtype and Δ*tarB* mutants, results are reported as the total CFUs estimated in small intestine homogenates, in single strain infections the Δ*tarB* mutant also shows a hypercolonization phenotype relative to wildtype (p = 0.02 two sample t-test on log transformed data). **D**) Competitions were carried out in mice after preincubation of the Δ*tarB* and parental strains in pond water for 4, 6 and 24 h. The Δ*tarB* mutant preincubated for 4 h in pond water has a fitness advantage over the parental strain, similar to competitions performed without pond water preincubation. Competitions performed after 6 h of preincubation show no significant trend. However, after 24 h of preincubation there is a reversal of the above phenotype with the parental strain having a significant advantage over the Δ*tarB* mutant (p<0.05, Wilcoxon signed rank test on log transformed data). Importantly, these strains do not show any difference in fitness during growth in LB after 24 h pond incubation.

Deletion of each sRNA was constructed in the genome and the mutants were competed against the fully virulent parental strain carrying a Δ*lacZ* marker. No significant difference in virulence was observed for the Δ*tarA* strain either when competed against the parental strain or a strain harboring *tarA* (promoter and *toxboxes* included) on a high-copy vector (figure 3A). It was previously reported that a Δ*tarA* mutant had a decreased fitness relative to its parental strain [25], however, those experiments were performed with a classical biotype strain of *V. cholerae*, and hence regulation by TarA may be less critical or perhaps is masked in the current pandemic El Tor biotype tested here.

In contrast the Δ*tarB* strain outcompetes the parental strain by a small but statistically significant factor of 1.6 (figure 3A) suggesting TarB is a negative regulator of virulence. The Δ*tarB* and complemented strains show no change in growth rate or cell yield in Luria-Bertani (LB) broth or in a minimal medium, nor a change in survival in pond water (figure S1).

To see if the negative effect on virulence could be complemented *in trans*, we competed a Δ*tarB* strain containing the sRNA with its own promoter cloned onto a low copy plasmid (p*tarB*) against a Δ*tarB* strain carrying empty vector (pMMB). The Δ*tarB* strain out-competed the complemented strain to an extent that exceeds out competition of the parental strain (figure 3A), which may be due to overexpression of TarB from p*tarB*. If expression of TarB is detrimental to colonization, as these data indicate, the plasmid carrying TarB may be selected against during the infection. To investigate this, small intestine homogenates were plated on LB agar and colonies were replica plated onto medium containing ampicillin, which selects for colonies containing the plasmid. Consistent with our hypothesis, the plasmid carrying TarB was lost more frequently than the empty plasmid (figure 3B). This was not the case during growth in LB in the absence of antibiotic selection (data not shown).

For further confirmation of the hypercolonization phenotype of the Δ*tarB* mutant, we performed single strain infections with the Δ*tarB* and wildtype strains (Figure 3C). Total colonization in these two strains indicated that, as seen in competition experiments, the Δ*tarB* mutant showed significant hypercolonization reflected by increased CFUs in the output.

The out-competition phenotype of the Δ*tarB* strain in infant mice and more drastic attenuated phenotype of the complemented Δ*tarB* strain suggest that TarB is deleterious to colonization of the small intestine. The model that TarB is positively regulated by the master virulence gene activator ToxT, yet functions as a negative regulator of virulence, is counterintuitive. To investigate this model further we performed competitions after incubation of the competing strains for varying times in filter sterilized pond water in an attempt to test the strains in a scenario more similar to a natural infection. After 4 hours (h) of incubation in pond water, the Δ*tarB* mutant retained its ability to outcompete the parental strain, but this phenotype was lost after 6 h of incubation in the pond (figure 3D). After 24 h of pond incubation, the parental now had a statistically significant advantage over the Δ*tarB* mutant when competed *in vivo*, but not when competed for *in vitro* growth in LB.

### Regulation of TarB

**Figure 4:**
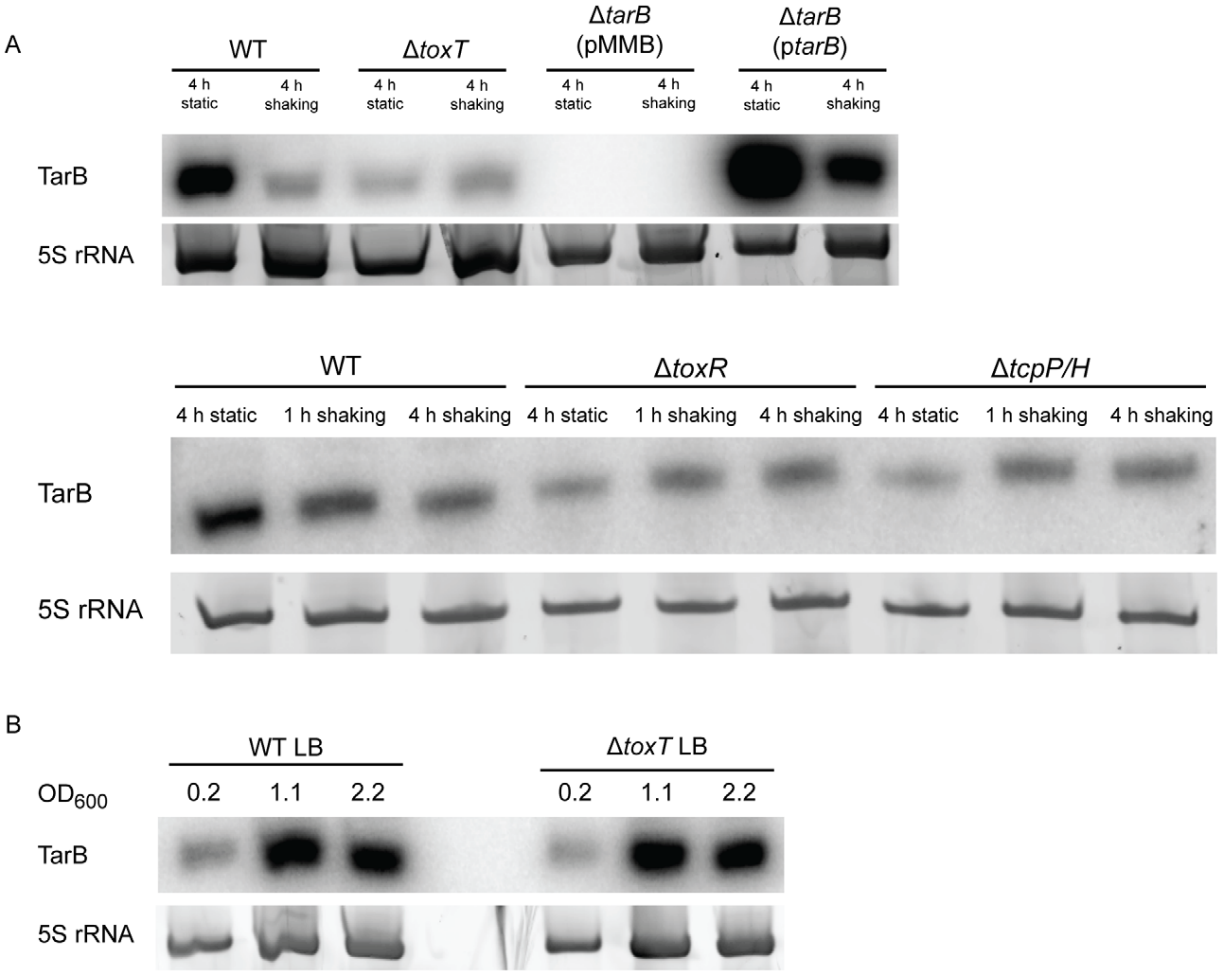
Northern blots of TarB during AKI induction and growth in LB. **A**) To determine the pattern of TarB expression during virulence factor inducing culture conditions (AKI induction), RNA samples taken after 4 h of static growth, 1 h of shaking growth and 4 h of shaking growth were blotted for the presence of TarB. TarB is upregulated during the static growth phase of AKI inductio. Upregulation of TarB was also dependent on *toxT*, *toxR* and *tcpP/H*. Expression from the complementation plasmid p*tarB* reveals that TarB is overexpressed from this plasmid 7–10 fold when adjusting for 5S rRNA loading, though the overall expression pattern of TarB remains the same. **B**) During growth in LB, *tarB* is upregulated upon entry into stationary phase, however, this upregulation is independent of ToxT.

To further investigate the ability of ToxT to control TarB expression, we measured expression of TarB under an *in vitro* virulence factor inducing condition, which is growth for 4 h static in AKI broth containing sodium bicarbonate followed by 4 h with aeration [28]. Expression of TarB is induced during the initial static phase of growth, but returns to background level after 4 h of growth with aeration (figure 4A, top panel). The initial induction is dependent on *toxT* as well as *toxR* and *tcpP/H* (figure 4A, bottom panel), which are genes upstream in the ToxR regulon that induce ToxT expression [3], [4], [29]. We also noted that TarB is overexpressed between 7–10 fold in the complemented strain, which is consistent with its *in vivo* phenotype being more dramatic then the parental strain in competitions with the Δ*tarB* mutant.

We also investigated the role of the RNA chaperone Hfq in TarB stability and action as many sRNAs that act in conjunction with Hfq are destabilized in its absence [30], [31]. To investigate expression from the TarB promoter we constructed a transcriptional fusion of a reduced half-life allele of GFP (GFP-ASV) [10], [32] to the TarB promoter. The fusion was used to measure activity of the TarB promoter during induction of ToxT from the pToxT plasmid in both Hfq^+^ and Hfq^−^ strains. In these same strains, steady state levels of TarB from a native copy of the gene were measured by northern blot. The results of these experiments are summarized in Figure S2 and indicate that Hfq likely does not play a role in stabilizing TarB or in its interaction with its target (described later).

Some basal expression of TarB is seen during culture in LB, which is greatly enhanced at the transition to stationary phase, however this increase is independent of ToxT (figure 4B). Enhanced expression of TarB during late exponential and stationary phase growth in LB broth and in the static phase of AKI growth (see above) may be related to oxygen tension in solution. To investigate the contribution of oxygen tension during AKI static growth to TarB expression we measured expression of ToxT, TcpF and CadC by qRT-PCR and TarB via the TarB-GFP fusion over the static growth period of AKI. The transcription factor CadC is activated by the LysR homologue AphB under low oxygen and low pH conditions [33], and its measurement is used here as a method of determining when the culture is undergoing those conditions. Additionally, AphB has been shown to be critical for activation of TcpP/H [34], [35], which in turn activates ToxT expression. The results of this experiment are summarized in figure S3. As measured against expression after two hours of static growth, expression of ToxT and TcpF have more or less reached maximum by 3 hours of static culture (Figure S3 A), though expression of TarB-GFP and CadC continue to rise, suggesting additional activation of the TarB and CadC promoters. Western blot for TcpF in the TcpF-FLAG fusion strain grown under the same conditions independently confirms this finding for TcpF (Figure S3 A), though OmpU could not be used as a loading control for this blot [36], so we did not carry out quantification of this blot. To investigate the contribution of anaerobiosis to expression of TarB, we prepared cultures of wildtype and Δ*toxT* strains in phosphate buffered LB media to prevent large alterations in pH and with glucose to support anaerobic growth [37]. These cultures were prepared in an anaerobic chamber and then grown either aerated in 2 mL or in sealed 10 mL cultures to approximately the same optical density, RNA extracted from these cultures was used in northern blots for TarB (Figure S3 B). The results show that anaerobic conditions do stimulate TarB expression independent of ToxT, though increases in expression of TarB in the wildtype culture were approximately twice as great when adjusting for loading, indicating that under anaerobic conditions, ToxT does drive some expression of TarB. Taken together these results suggest that anaerobic conditions activate TarB expression possibly through the action of AphB.

**Figure 5:**
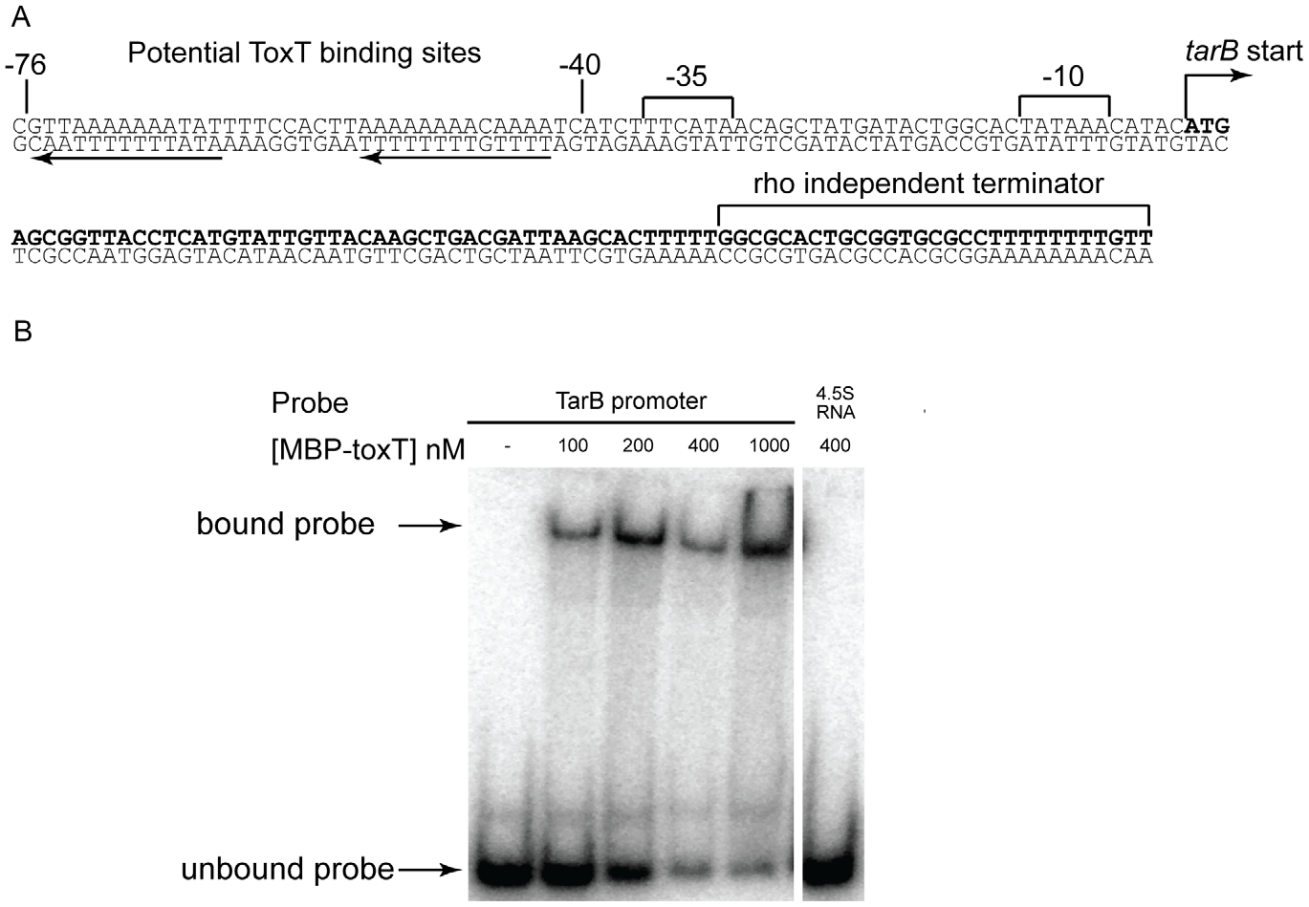
Sequence of *tarB* and ToxT binding sites within promoter region. **A**) Sequence of *tarB* as determined by the sRNA deep sequencing experiment. Direct repeats of the putative ToxT binding sequence are highlighted by the black arrows. **B**) Electrophoretic mobility shift assays using uncleaved MBP-ToxT fusion protein and the sequence 100 bp upstream of the *tarB* transcriptional start site as a probe. A PCR product of the same size consisting of the sequence of *ffh*, the gene encoding the 4.5S RNA, was used as a negative control probe.

The sequence upstream of the predicted TarB start site was investigated and revealed putative −10 and −35 sequences, as well as a direct repeat of putative *toxboxes* (figure 5A). The 3′ end of TarB determined by deep sequencing corresponded to the poly-U tract of a Rho independent terminator. The *toxboxes* upstream of *tarB* are arranged in similar fashion to those upstream of the virulence gene *tcpA* [7]. To confirm binding of ToxT to this site, a DNA probe consisting of basepairs −100 to +1 relative to the predicted transcription start site was assayed for ToxT binding by gel shift assay. ToxT bound to this region with an affinity within the range of other reported *toxboxes* [38], but not to a non-specific probe of similar length consisting of a PCR product of the 4.5S RNA sequence (figure 5B).

### *tcpF* mRNA is a target of the TarB sRNA

**Figure 6:**
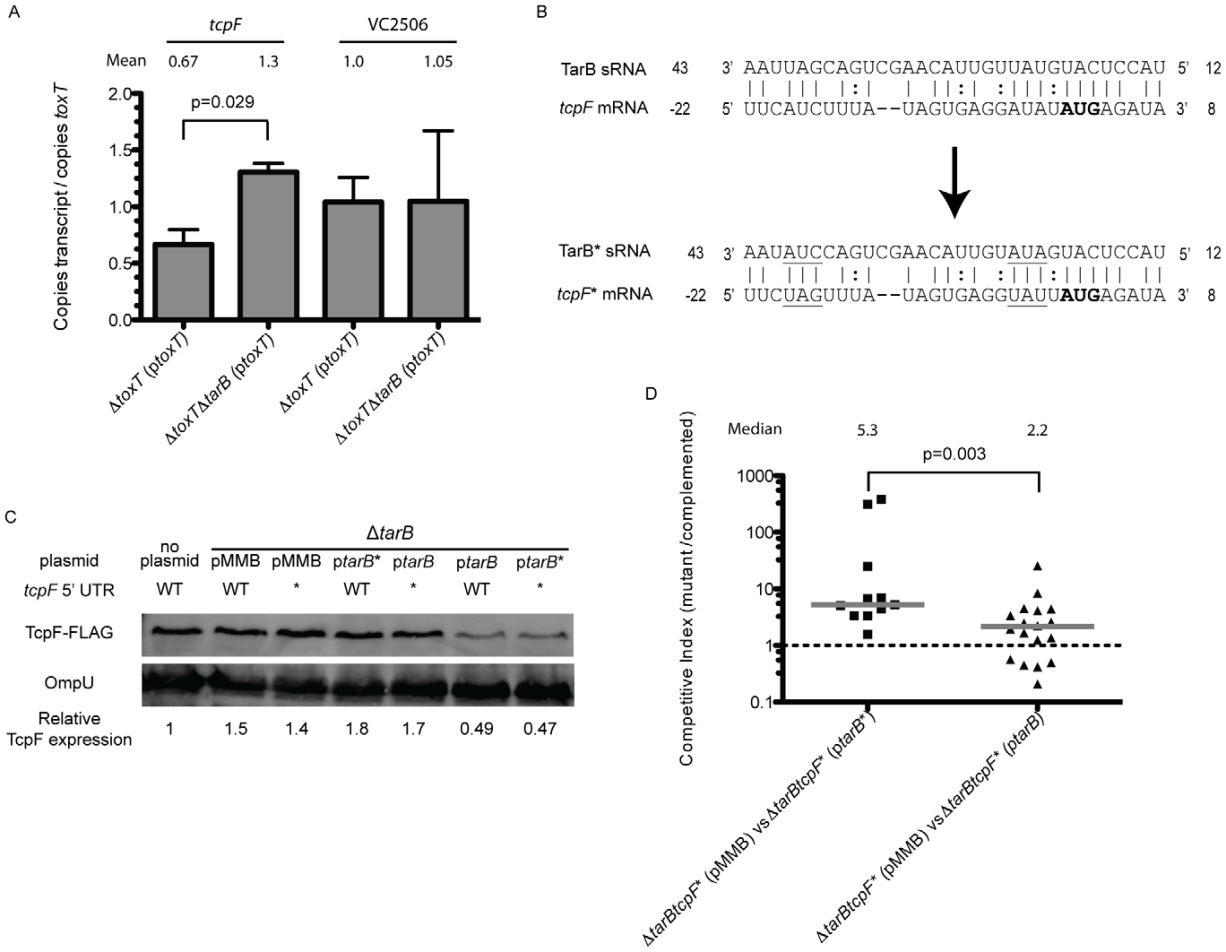
TarB interaction with the 5′ UTR of *tcpF*. **A**) Quantitative reverse transcription PCR was carried out on RNA extracted from strains expressing ToxT from an arabinose inducible promoter. Because the extent of *toxT* induction varied between experiments, transcript levels were normalized to *toxT* transcript. The Δ*tarB* mutant showed a significant enhancement of 2-fold in *tcpF* transcript relative to wild type over the course of 4 independent experiments (Mann-Whitney U test), the level of the other predicted target (VC2506) did not change. **B**) Predicted base pairing interaction between TarB and the 5′ UTR of *tcpF*. The start codon of TcpF is highlighted in bold, the numbering of the *tcpF* transcript is relative to the start of translation, numbering of the TarB transcript is relative to the start of transcription. The mutations made to generate tcpF* and tarB* are underlined. **C**) A fusion of the FLAG peptide to the C-terminus of TcpF was generated to follow TcpF expression by western blot. At the 4 h static time point of AKI induction a band corresponding to the molecular weight of TcpF-FLAG was detected with the anti-FLAG antibody. Blots were then stripped and re-blotted with anti-OmpU antibodies to serve as a loading control. Fluorescence measurements of TcpF-FLAG bands were divided by measurements of OmpU bands. Results are shown for the wild type strain without plasmid (first column) and for the Δ*tarB* strains containing the wild type or mutated TarB cloned on the pMMB plasmid and either the wild type or mutated *tcpF* 5′ UTR (tcpF*) chromosomal allele (columns 2–7). Expression values are standardized to TcpF-FLAG measurements adjusted for loading in the wildtype strain. **D**) Competitions in infant mice between Δ*tarB* strains carrying the tcpF* allele complemented with p*tarB* or ptarB* against the same strains carrying pMMB67EH alone. The strain complemented with ptarB* shows decreased colonization relative to the empty vector strain. When complemented with p*tarB* the competitive index is closer to 1 (Mann-Whitney U test).

We next wanted to determine the target(s) of TarB that were responsible for the observed negative role of TarB in virulence. Nineteen putative mRNA targets were identified using the program targetRNA [39], which searches for complementarity between the query sRNA and the 5′ untranslated region (UTR) of mRNAs of annotated ORFs within a given genome. To validate putative targets, we looked for changes in the steady-state level of the candidate mRNAs using quantitative reverse transcription PCR (qRT-PCR) on total RNA from TarB^+^ and *ΔtarB* strains both over-expressing ToxT. Of the six putative targets we selected for further analysis only two, *tcpF* and VC2506, had any detectable expression under the conditions tested. When levels of the potential target transcripts were normalized to *toxT* transcript levels, a significant difference between the TarB^+^ and *ΔtarB* strains was revealed for the *tcpF* mRNA but not for VC2506 (figure 6A). The observed increase in *tcpF* mRNA in the *ΔtarB* background suggests that TarB negatively regulates *tcpF*, which would be consistent with the negative role of TarB in virulence.

To determine if TarB similarly affects TcpF protein expression level, we generated a C-terminal FLAG tag fusion to TcpF in the genome to measure expression by western blot after AKI induction. We also generated two sets of three point mutations each within the predicted region of complementarity between TarB and the 5′ UTR of *tcpF*, yielding tcpF* and tarB* alleles. These mutations are underlined in figure 6B. Because the *tcpF* and *tcpE* ORFs are very close together, there is some overlap between the coding sequence of *tcpE* and the 5′ UTR of *tcpF*, however the substitutions made do not affect the amino acid coding sequence of the upstream gene *tcpE* nor do they alter the Shine-Dalgarno sequence of *tcpF*. Moreover, the mutations were designed to preserve GC content of the region altered. Either set of mutations present alone (tarB* or tcpF*) would be predicted to disrupt the interaction between TarB and the *tcpF* 5′ UTR while the presence of both is compensatory and would be predicted to restore the interaction.

A strain deleted for *tarB* was then used as the parent strain to construct derivatives having either the *tcpF*-FLAG or tcpF*-FLAG allele. These two derivatives were then complemented with either p*tarB*, ptarB* or empty vector (pMMB). These six strains along with the wild type strain carrying the TcpF-FLAG fusion were grown through the static culture phase of an AKI induction and were western blotted to measure TcpF-FLAG expression. The blots were then stripped and probed for OmpU, which is not regulated by ToxT [40], to serve as a loading control. Compared to the wild type strain (figure 6C, first column) the Δ*tarB* and ΔtarB tcpF* strains carrying the empty vector showed elevated TcpF levels. When the Δ*tarB* and ΔtarB tcpF* strains were complemented with ptarB* and p*tarB*, respectively, levels of TcpF remain largely unchanged, indicating that when either the *tcpF* mRNA or *tarB* sRNA are mutated, no interaction can take place and these strains show expression of TcpF similar to the Δ*tarB* mutant. However, when the Δ*tarB* and ΔtarB tcpF* strains were complemented with p*tarB* and ptarB*, respectively, to observe affects of the wild type or compensatory interaction when the sRNA is overexpressed, the levels of TcpF drop substantially. Six replicates of this experiment were performed and reveal that statistically significant drops in expression of TcpF occur only in strains containing either the wildtype TcpF target sequence complemented with wildtype TarB or strains in which the target sequence and sRNA have compensatory mutations (Figure S4). When these strains were blotted after the aeration growth phase of AKI induction, no differences in TcpF expression were visible (data not shown), which would be expected given the up regulation of *tarB* during the static phase but return to basal level of expression during the aeration phase of AKI induction.

To determine if the interaction of TarB with the 5′ UTR of *tcpF* was responsible for the phenotype in mice, competitions were carried out using tcpF* strain derivatives. Competition of the ΔtarB tcpF*(ptarB*) strain against the same strain carrying empty vector yielded the expected result of out-competition by the latter strain, which lacks tarB* (figure 6D). Competition of the ΔtarB tcpF*(p*tarB*) strain against the same strain with vector alone yielded a competitive index that was significantly closer to one, which is expected since neither strain should have an interaction between sRNA and target. The difference between the two competitive indices was highly significant (p<0.003).

To determine if the pond water-incubation phenotype of the Δ*tarB* mutant was related to expression of TcpF or TarB in this environment we carried out experiments to measure TarB and TcpF levels over the course of pond water incubation, the results of these experiments are summarized in Figure S5. TcpF expression was followed through the course of pond water incubation via the C-terminal FLAG fusion in both the wildtype and Δ*tarB* backgrounds by anti-FLAG western blot. The results indicate that the wildtype and Δ*tarB* mutant show similar levels of TcpF expression initially, however, over the course of pond incubation, TcpF levels drop in the wildtype strain, but not the Δ*tarB* strain. Transcription of TarB, as measured by production of GFP from the TarB promoter-GFP fusion indicates that levels of TarB expression do not change dramatically over the course of pond water incubation. Northern blots for TarB expression over the course of pond water incubation suggest that TarB steady state levels drop (data not shown), but this may be due to the observed wholesale degradation of RNAafter increasing time of incubation in pond water, such that accurate measurements of TarB expression via northern blot are not possible. These results indicate that while TarB expression levels do not vary dramatically over the course of pond water incubation, TcpF protein levels do drop, and this drop was absent in the Δ*tarB* mutant. This enhanced TcpF expression in the Δ*tarB* mutant may contribute to the phenotype of the Δ*tarB* mutant *in vivo* after pond water incubation, as over expression of TcpF in pond water would contribute to metabolic drain prior to infection.

## Discussion

Deep sequencing has allowed the interrogation of processes in bacteria with unprecedented detail. Here we used two complementary approaches, deep sequencing of cloned sRNAs and ToxT-bound DNA fragments, to identify ToxT-regulated sRNAs. The number of previously estimated ToxT binding sites in the *V. cholerae* genome was between 17 and 20 [9], [27]. We have now uncovered what may be a greatly expanded set of targets for ToxT to coordinate expression of protein coding genes as well as sRNAs. The results of the pulldown experiment returned regions of a few hundred basepairs in length that were enriched and many predicted sites are overlapping, which is due to the size range of the fragments used in the pulldown and the automated analysis of the pulldown data. Although many of these sites remain to be validated we are confident in proposing that the ToxR regulon encompasses many more transcripts, both protein coding and otherwise, than was previously thought.

The results of the sRNA deep sequencing reveal the method to be exquisitely sensitive. Because of our exclusion of larger RNA transcripts and depletion of tRNA and 5S RNA in the sRNA size range and the use of Illumina massively parallel sequencing technology we have achieved tremendous depth of coverage of potential sRNA genes in *V. cholerae* [16]. Transcripts represented by ∼40 or more reads could be detected by northern blot (this study and data not shown). However, transcripts represented by fewer than ∼40 reads, which may represent low abundance sRNAs, are difficult or impossible to detect by northern blot and other methods such as qRT-PCR are needed for independent validation. Of the 18 candidate ToxT-regulated sRNAs we report here, 11 (including *tarB*) were not identified as putative sRNAs in previous sequencing experiments or bioinformatics-based approaches to sRNA discovery [16], [41], displaying the depth of information that can be gained with high throughput sequencing technologies and the conditional expression of sRNAs. In comparison to other methods of sRNA discovery, our approach has the advantage of being targeted in its search for ToxT-regulated sRNAs but unbiased in its identification of sRNAs. Approaches utilizing RNA binding proteins such as Hfq [42], [43], are not exhaustive as the sRNA we report here likely does not interact with Hfq, though those methods do have the potential to identify mRNA targets as well as sRNAs. Additionally, this approach benefits from the vast strides made in high throughput sequencing recently which generates far more depth of data then microarray based methods [44], including exact 3′ and 5′ ends and unbiased coverage of positive and negative strand sRNAs. Keeping the latter in mind, this approach can also identify many potential sense and anti-sense sRNAs [16] overlapping with protein coding genes although these potential sRNAs are not discussed here.

In this study we identified a new sRNA member of the ToxR regulon that fine-tunes expression of a virulence factor also within the ToxR regulon, thus adding a new facet to the elaborate virulence gene regulation program in *V. cholerae*. However, when placed in the larger context of *V. cholerae* pathogenesis, it is not entirely clear why a repressor of an essential virulence factor would be produced at the same time as the virulence factor it negatively regulates. The answer may lie in the biphasic nature of *V. cholerae* gene expression during intestinal colonization [10], [11]. The initial induction of virulence factors requires ToxR/S- and TcpP/H-dependent ToxT expression in the intestinal lumen. This is followed by a more robust activation of the TCP and CTX operons closer to the epithelial surface of the small intestine, driven by a positive feedback loop in ToxT expression that is thought to activated in part by the presence of bicarbonate [28], [45].

During AKI induction *in vitro* in the absence of bicarbonate, ToxT production is stimulated during static growth but the transition to aerated growth is required for CTX production [46]. All experiments reported here included bicarbonate in the medium over the course of the experiment, which is sufficient to cause CTX production even during static growth [28], [47]. Research done on the contribution of anaerobiosis to virulence gene expression in *V. cholerae* El Tor isolates has shown stimulation of VPI gene products [48], and that the AphB protein, which functions upstream of *tcpP/H*, is active primarily at low oxygen tension and low pH [33]. Since TarB expression is greatest during the static phase of AKI induction, but repressed during aerated growth even though bicarbonate had been added to induce CTX and TCP expression prior to aeration, it is tempting to speculate that TarB expression is enhanced in microaerobic conditions. The experiments we performed under anaerobic conditions also suggest that oxygen plays a role in TarB expression, though it may be only one of a host of signals, which act on TarB *in vivo*. TarB's function under low oxygen tension could be to repress TcpF expression prior to penetration of the mucous barrier of the small intestine. Upon reaching the epithelial surface, the higher oxygen tension would contribute to reduced TarB expression, allowing TcpF to be fully expressed. This would fit with the proposed role of TcpF in colonization of the epithelium [49]. The intestinal brush border is a highly vascular structure, commensurate with its role in absorbing nutrients, and it would not be unreasonable to speculate that the lumenal space adjacent to it would have greater oxygen tension then the luminal fluid. The actual oxygen tension of the small intestine may be quite low as oxygen requiring luciferase reporter systems in bacteria do not function in the small intestine [50], [51]. However, to the best of our knowledge, oxygen measurements at the brush border have not been reported.

Other possible factors responsible for controlling TarB expression could be entry into stationary phase, as increased TarB expression is observed in *V. cholerae* grown in LB broth to late exponential and stationary phase. Also, during AKI induction, 4 hours of growth in static culture corresponds with entry into stationary phase [46]. Stationary phase regulation of TarB may also occur via an alternative sigma factor as was observed for the sRNA VrrA [20], or possibly via CRP-cAMP mediated repression as carbon sources become depleted [35].

Coordination of TcpF expression by TarB appears to have a positive effect on colonization if the bacteria are coming from a resource poor environment, such as contaminated pond water, and even then, the differences in colonization efficiency of the Δ*tarB* mutant are quite small. In contrast, if the bacteria are grown in a rich medium prior to infection, overexpression of TcpF in the Δ*tarB* mutant appears to be beneficial. The reasons for this may relate to the details of the experimental system used here, wherein immunologically naïve infant mice are used as a host. In contrast, in nature many hosts in endemic areas will have some level of pre-existing immunity, and may harbor anti-TcpF antibodies as TcpF is a known antigenic protein [49]. It is possible that tight repression of TcpF provides a more pronounced fitness advantage in nature under different conditions then those used here, which would explain TarB's presence among all sequenced isolates of toxigenic *V. cholerae* (data not shown). Further insight into the functional role of TcpF in colonization may shed more light on the necessity of the TarB-mediated post-transcriptional regulation observed here.

## Materials and Methods

### Ethics statement

All animal experiments were done in accordance with NIH guidelines, the Animal Welfare Act and US federal law. The experimental protocol using animals was approved by Tuft University School of Medicine's Institutional Animal Care and Use Committee. All animals were housed in a centralized and AAALAC-accredited research animal facility that is fully staffed with trained husbandry, technical, and veterinary personnel.

### Bacterial growth conditions

*V. cholerae* O1 serogroup El Tor biotype isolate E7946 and derivatives were grown at 37°C in LB broth with aeration. For AKI induction, strains were grown in AKI broth (1.5% peptone, 0.4% yeast extract, 0.5% NaCl, 0.3% NaCHO_3_) statically for 4 h at 37°C followed by aeration for 4 h 37°C. To induce expression of cloned genes on plasmids, arabinose was added to 0.04% upon reaching mid-exponential phase (optical density at 600 nm [OD] = 0.3). All DNA manipulations were done in *E. coli* DH5α or derivatives with plasmids maintained with the appropriate antibiotics.

### Strain construction

All PCR reactions were carried out with EasyA polymerase according to the manufacturer's specifications using the indicated primers, the sequences of which can be found in table S1. The descriptions of all plasmids used in this study are included in table S2.

Plasmids pToxT and pToxT ΔHLH plasmids we constructed by PCR amplification of the *toxT* ORF including native RBS from gDNA from either wildtype *V. cholera* E6749 or an E6749 strain carrying an internal deletion of the helix-loop-helix DNA binding domain [11] using primers NcoI_ToxT_F and XbaI_ToxT_R. This PCR product was then cloned into the NcoI and XbaI sites of the pBAD24 plasmid [52] to allow expression of ToxT upon addition of L-arabinose. Unmarked deletions of chromosomal genes were constructed by SOE PCR introduced using a derivative of the pCVD442 allelic exchange vector, pCVD442-lac which contains the pUC19 LacZ gene and MCS, as described [53].

Point mutations in the *tarB* gene were generated by SOE PCR using primers xbaI_TarB comp_F, TarB_mut_R1 and TarB_mut_F2 and SacI_TarB_comp_R, using E6749 genomic DNA as template. PCR products were mixed in a one to one ratio, and added to a PCR reaction run for 25 cycles at an annealing temperature of 50°C without primers and the mutated sRNA sequence plus promoter were amplified with XbaI_TarB_comp_F and SacI_TarB_comp_R which contain SacI and XbaI restriction sites which were subsequently used for cloning into pMMB67EH to generate ptarB*. The wildtype complementation vector p*tarB* was generated by cloning a PCR product generated using XbaI_TarB_comp_F and SacI_TarB_comp_R primers and genomic DNA as a template.

Point mutations in the *tcpF* 5′ UTR were also generated by SOE PCR using primers XbaI_TcpF_mut_F1, TcpF_mut_R1, TcpF_mut_R2 and XbaI_TcpF_mut_R2 using an identical procedure as above. The final ∼2 kb product containing the mutated *tcpF* 5′ UTR sequence which was subsequently cloned into the XbaI site of the pCVD442-lac vector which was then mated into strains of interest. Double crossovers were selected on 10% sucrose plates. Individual double crossovers were screened for the mutated sequences by sequencing with the TcpF seq primer and the XbaI_TarB_comp_F primer and confirming double crossover by streaking on 10% sucrose as well as ampicillin containing plates to ensure sucrose resistance and ampicillin sensitivity.

C-terminal FLAG fusions to TcpF were generated by amplification of the C-terminal 346 bp using the TcpF_qt_F primer and the TcpF-FLAG_R primer to add the FLAG amino acid sequence [54], this product was subcloned into Topo pCR2.1 (Invitrogen). The resulting plasmid was cut using KpnI and EcoRV and the insert containing the C terminus of TcpF with the FLAG fusion was cloned into a modified pGP704 suicide vector [55] which contains a chloramphenicol resistance drug marker in place of an ampicillin marker (pGP704cat). This construct was then mated into strains of interest and single crossovers were selected for on chloramphenicol plates at 2 µg/mL. Proper insertions were confirmed by PCR using the TcpF-FLAG reverse primer and TcpF seq forward primer. A merodiploid strain was constructed by plasmid integration resulting in the placement of GFP(ASV) under the control of one copy of the TarB promoter followed by the native TarB locus downstream of the integrated plasmid sequence. The plasmid borne fusion was generated by amplifying the +3 to −376 positions in the TarB promoter from E6749 genomic DNA using primers TarB_F and TarB_-300_R and subcloning the product into pCR2.1 yielding p*tarB-300*. GFP was amplified from pGfpmut3.1 plasmid (Clonetech) using primers Fgfp2 and Rgfp2 which adds a ribosomal binding site and SacI site at the 5′ end and the destabilizing (ASV) [32]C terminal amino acids and a SmaI site at the 3′ end. The GFP(ASV) PCR product was cloned in a triple ligation with the SacI/EcoRV fragment from p*tarB-300* into pGP704cat digested with SmaI to generate the transcriptional fusion. The resulting plasmid (pTarB-GFP) was mated into E6749 strains and single crossovers were selected on chloramphenicol and confirmed by PCR using primers Rgfp2 and XbaI_ΔTarB_R2.

### sRNA deep sequencing

Single colonies of strain AC3763 (Δ*toxT*) transformed with either pToxT or pToxTΔHLH plasmids were picked and grown in LB broth containing streptomycin and ampicillin both at 100 µg/mL overnight. Strains were back diluted from overnight cultures to an OD of 0.03 in 200 mL LB supplemented with streptomycin and ampicillin both at 100 µg/mL and were grown with aeration at 37°C until the strains reached mid-exponential phase (OD = 0.3). Arabinose was then added to 0.04% to induce expression of *toxT* alleles from pToxT plasmids, and induction was allowed to proceed for 20 minutes prior to RNA extraction. Total RNA was purified from the bulk culture by phenol/chloroform extraction and isopropanol precipitation. Cloning and sequencing of sRNA was carried out as previously described [16], sequences of the micro RNA cloning linkers (IDT) used are included in table S4. In order to further decrease tRNA and 5S rRNA in the final sequenced products, the depletion step described in the previously published procedure was carried out twice with the addition of an oligo targeting the serGCC tRNA (5′-GCGGTGAGTGAGAGATTCGAACTCTC-3′). The final cDNA products were prepared for Illumina Genome Analyzer II sequencing using Illumina primers 1a, 1b and 1c (table S1) for the first 10 cycles of PCR, followed by gel purification and Illumina primers 2a and 2b (table S1) for the final 4 cycles of PCR followed by PCR clean up (Stratagene). Final products were run on a Bioanalyzer High-Sensitivity DNA chip (Agilent) prior to Illumina sequencing to normalize loading of the two samples and ensure quality of the libraries. The libraries were pooled and placed on one lane of an Illumina Genome Analyzer IIx paired-end sequencing run at Tufts University Core Facility. Briefly, a paired-end sequencing run sequences both the 5′ and 3′ end of every DNA molecule attached to the flowcell. The first read is downstream of linker 1 and the second read is downstream of linker 2 (ToxT library) or linker 3 (ΔHLH library) so that for every pair, the directionality of the original RNA molecule could be determined. Sequence reads were trimmed to remove linker sequences and filtered so that 100% of the sequenced bases in each read had a minimum quality score of 5 (base call accuracy at least 68%). Reads were aligned to the O1 biovar N16961 genome (NCBI Accession Nos. NC_002505, NC_002506) using Bowtie (http://bowtie-bio.sourceforge.net). Reads matching rRNA or tRNA regions were removed from the alignment, leaving 1,062,048 reads in the ToxTΔHLH library and 2,212,216 reads in the ToxT library. Unique transcripts totaled 6,815 for ToxTΔHLH and 27,787 for ToxT. The alignments were then processed to generate a library of clustered transcripts using the method previously described [16]. This resulted in 3,309 clusters for the ToxTΔHLH library and 12,534 clusters for ToxT library. Clustered reads were output into “gff” format and viewed using GenomeView (http://genomeview.org). The number of reads in sRNA clusters were normalized by dividing the number of reads in that cluster by the ratio of MtlS reads in that library to total MtlS reads. For example normalized reads_ToxT_ = cluster reads_ToxT_/(MtlS_ToxT_/(MtlS_ToxT_+MtlS_ToxTΔHLH_)).

### ToxT overexpression and purification

*E. coli* strain BL21(DE3) was transformed with the plasmid pMAL-TEV-His-thr-ToxT (table s3). The resulting strain was grown on LB agar plates containing ampicillin and a single colony was picked for growth of a 4 mL overnight culture. The overnight culture was used to inoculate 1 L LB broth containing ampicillin at 100 µg/mL and was grown with aeration at 37°C. Transcription was induced once the culture had reached exponential phase (OD = 0.5–1) by addition of IPTG to 1 mM. Induction was allowed to proceed shaking at 20°C for 16 h, after which, cell pellets were collected by centrifugation and resuspended in 20 mL lysis buffer (20 mM Tris-HCl pH 8, 2 mM DTT, 1 mM EDTA, 250 mM NaCl) plus Complete protease inhibitors (Roche). Cell pellets were lysed and the lysate was cleared by centrifugation at 18,000 rpm in a SS34 rotor.

The cleared lysate was then applied to a 5 mL dextrin MBPtrap column (GE Life sciences). The column was washed with lysis buffer followed by elution with MBP elution buffer (as lysis buffer, +1 mM maltose). The elution fractions were subsequently diluted 10-fold with buffer QB1A (20 mM Tris-HCl pH 8.0, 1 mM DTT) and applied to an 8 mL Source15Q anion exchange column equilibrated in QB1A. The protein was eluted using a 0 to 20% gradient of QB1B (20 mM Tris-HCl pH 8.0, 1 M NaCl, 1 mM DTT) developed over 25 column volumes. The peak fractions were diluted 5-fold in SB1A buffer (25 mM phosphate buffer pH 6.0, 1 mM DTT) and applied to a 8 mL Source15S cation exchange column equilibrated in SB1A. The protein was eluted using a 15 to 35% gradient QB1B (25 mM phosphate buffer pH 6.0, 1 mM DTT, 1M NaCl), which resulted in two peaks, the second peak was known to be a soluble aggregate and was discarded. The initial peak was split into two aliquots, one of which was applied to a Superose 12 gel filtration column in EMSA buffer (10 mM Tris-HCl pH 7.5, 200 mM KCl, 10 mM βME) for use in mobility shift assays, the other aliquot was cleaved with TEV protease overnight at 4°C and subsequently diluted 5-fold in SB1A and applied to a 2 mL Source15S cation exchange column to separate His-ToxT from the cleaved MBP fusion protein. His-ToxT was eluted from this column with a 35 to 100% gradient of SB1B developed over 12 column volumes. Finally, His-ToxT peak fractions were applied to a Superdex 75 gel filtration column in EMSA buffer. These final steps did leave a small amount of TEV protease in the final purified product.

### Affinity purification of ToxT binding sequences

Genomic libraries were prepared by centrifuging 10 mL of overnight growth of wild type (AC53) *V. cholerae*, washing 2× with TBS and resuspending in 5 mL TBS. To generate gDNA fragment sizes of 300 to 1,000 bp, the cell pellet was subjected to four 30 second sonication cycles on ice using a sonicator micro tip (Branson); each sonication cycle was separated by a 30 second incubation on ice. After sonication, RNAase A was added to a concentration of 2 µg/mL, the samples were incubated at 37°C for 20 min to allow for degradation of RNA. DNA was purified with 2 rounds of extraction with citrate buffered phenol∶chloroform (Ambion) followed by a final extraction with chloroform only and then concentrated by ethanol precipitation. Fragmented DNA was used to prepare three different bar-coded libraries using adapters BC1a/BC1b, BC2a/BC2b and BC3a/BC3b (table S4) as described [26]. For the final amplification and purification of bar-coded libraries, ten PCR reactions were done using linkered and size selected gDNA as template using primers Olj 139 and 140 and EasyA polymerase (Stratagene). PCR conditions were as follows, denaturation for 5 minutes at 95°C, annealing for 30 seconds at 65°C, elongation for 30 seconds at 72°C, cycling back to denaturation at 95°C for 30 seconds for 15 cycles after which reactions were pooled and incubated with 50 µL ExoSAP-IT (USB) at 37°C for 1 h. Final purification of libraries was carried out by phenol∶chloroform extraction and ethanol precipitation and resuspension of libraries in 100 µL deionized water.

Binding reactions contained 15 µg bar-coded DNA library in a total volume of 250 µL with 200 nM purified His6-tagged ToxT purified as above or with His6-tagged TEV protease in EMSA buffer with 10 µg/mL sheared salmon sperm DNA, 0.3 mg/mL BSA and 10% glycerol. Reactions were allowed to incubate with gentle mixing at 37°C for 1 h, after which the reaction was added to a microcentrifuge spin column (Pierce) packed with a 50 µL bead volume column of HisPur cobalt resin (Pierce) that had been equilibrated in the above buffer. The reaction was allowed to bind to the column by mixing gently at 37°C for 1 h. Flow through was then collected by spinning the column in a microcentrifuge at 3,000× g for 1 minute. The column was washed 3× by gentle resuspension of the bead volume in 250 µL of EMSA buffer with the above additions, followed by centrifugation. The column was washed an additional 3× as above, but in EMSA buffer only. After the final wash, the bead volume was resuspended in 10 mM Tris-HCl pH 8 and boiled for 5 minutes and allowed to cool to room temperature, then incubated with proteinase K (5 µg/ml) for 30 minutes at 65°C, followed by boiling for 5 minutes. After centrifugation for 1 min at 3,000× g, the resulting 100 µl of the supernatant fluid was purified by using a PCR purification kit (Qiagen) and then subjected to 10 cycles of PCR amplification with primers Olj139 and Olj140, repurified, quantified on the Bioanalyzer high sensitivity DNA chip (Agilent), and subjected to deep sequencing, along with aliquots of the input libraries prior to pulldown, using the Illumina Genome Analyzer II on the paired end setting.

Reads from the Illumina libraries were aligned to the N16961 genome. Sequence alignment and assembly were performed as described above. After alignment, reads that did not match the genome were discarded and the sets were normalized so that each set contained the same number of reads. Alignment positions were shifted by half their insert length as determined by each mapped pair, giving the center position of each sequenced DNA molecule. These positions were then tabulated and used to generate a coverage map of the genome using a rolling average with window size of 35 bases. Coverage maps were generated for every sample. For each genomic DNA and corresponding pulldown sample, an enrichment map was created, which represented the ratio of the values from the pulldown sample over that of the genomic DNA sample. Enrichment maps were then scanned to identify regions that had more than 3× the average coverage for more than 100 consecutive positions. The false discovery rate (FDR) was then calculated by performing the same analysis with the control and pulldown samples switched. At 3× coverage, the FDR was 0.03 and 199 enriched sites were identified totaled between the libraries, of which 67 were observed in both replicates. Significance of each enriched region was assessed using two methods [56]. First, the number of reads in that region in the control sample was used to generate a Poisson distribution. This was then used to assess the probability of the same number of reads occurring in the pulldown sample. Using this method, all regions identified had a p-value of <1×10^−98^. Second, a Z-score was found by comparing the proportion of tags in the control sample to that in the pulldown. All of the regions identified had a significant difference in the proportion of tags counted between the control and pulldown samples, with z-scores >7.7. The nucleotide sequences from the overlapping set were used as a training set for finding motifs using MEME 4.1.0. We allowed MEME to find motifs that occurred at least one time in each fragment. The motif reported in figure 1 panel C is the lowest E-value motif for the 67 sites combined in both libraries.

### Mobility shift assays

Primers TarB promoter R and TarB promoter F were used to amplify the upstream 100 bp of TarB, predicted to contain promoter elements and ToxT binding sites to serve as a probe in the mobility shift assay. The PCR product was purified (Stratagene) and 3.3 pmoles was end-labeled using T4 Polynucleotide Kinase (NEB) and ^32^P γ-ATP according to the manufactures instructions, and then purified using a Performa DTR spin cartridge (Edge Biosciences). A negative control probe of similar size consisting of 4.5S RNA sequence was prepared in parallel. The binding reaction occurred in 20 µL with 3 nM labeled probe and varying concentrations of purified MBP-his-thr-ToxT in EMSA with 10 mM 10 µg/mL sheared salmon sperm DNA, 6 µg/mL BSA, 10% glycerol and 0.002% Orange G dye added. Binding was allowed to occur for 30 minutes at 30°C followed by loading of the entire reaction onto a 5% TBE-Polyacrylamide gel, which was then run at 100 V for 60 minutes. The gel was then used to directly expose a phosphor screen and the image was read on a FLA-9000IR using the IP setting.

### AKI induction experiments

For AKI induction experiments, strains were grown overnight with aeration at 37°C in LB broth containing streptomycin at 100 µg/mL, ampicillin at 50 µg/mL (excluded in the case of the TcpF C-FLAG integration in the wild type background and TarB-GFP strains without plasmid) and chloramphenicol at 2 µg/mL. Overnight cultures were then diluted into prewarmed AKI media [47] containing 0.3% NaHCO_3_ and ampicillin at 50 µg/mL (again excluded for the wild type background strain and TarB-GFP fusions) to an OD of 0.01. Strains were grown statically in an incubator at 37C for the indicated times at which culture aliquots were removed for analysis. After 4 hours of static growth, cultures were split into 1 mL aliquots and grown shaking at 37C for 4 hours.

### Anaerobic growth

For anaerobic growth experiments, overnight cultures were prepared by inoculation of strains into phosphate buffered LB media containing 60 mM K_2_HPO_4_, 33 mM KH_2_PO_4_, 0.5% glucose and 100 µg/mL streptomycin. These cultures were grown overnight in an anaerobic chamber and used to subsequently inoculate either 2 mL aerated cultures or 10 mL cultures in sealed tubes prepared in the anaerobic chamber to an OD of approximately 0.01. Aerobic and anaerobic cultures were then grown in parallel in a shaking 37C incubator to approximately the same OD and snap frozen on liquid nitrogen and subsequently used for RNA extraction and northern blots. For each culture the pH of the media was measured after growth was recorded and ranged between 6.3 and 6.5 for anaerobic cultures and 6.7 to 6.8 for aerobically grown cultures.

### Pond water incubations

For pond water incubation experiments, strains were grown overnight on M9 minimal media+glucose plates containing the proper antibiotics. Overnight growth was resuspended in saline and washed twice. After the final wash, strains were resuspended in filter-sterilized pond water and inoculated into 2 mL culture tubes of filter sterilized pond water to an OD of 0.1 and incubated shaking at 37°C for the indicated times. At those times, culture aliquots were prepared either for western blot by centrifugation followed by resuspension in sample buffer and boiling or diluted to a density of 1×10^3^/µL as measured by OD for mouse infections.

### ToxT induction experiments

Experiments involving induction of ToxT from the arabinose inducible plasmid were carried out similarly to those used in sRNA sequencing experiments. Overnight cultures of the indicated strains were grown at 37°C overnight in LB containing the appropriate antibiotics. Overnight cultures were then diluted to an OD of 0.03 in 25 mL of the same media and allowed to grow shaking at 37°C. Once cultures reached mid-exponential phase (OD = 0.3), arabinose was added to a final concentration of 0.04% and induction was allowed to proceed for 1 h with 2 mL aliquots of culture taken at the indicated times and either spun down for western blot analysis or snap frozen in liquid nitrogen for RNA extraction later.

### Northern blots

Between 2.5–10 µg of total RNA purified using the Ambion mirVana kit from the indicated cultures was run on 10% TBE-urea polyacrylamide gels. Prior to transfer, gels were stained with GelStar (Invitrogen) and scanned on the FLA-9000IR (Fuji) to assess total RNA loading in each well and to use for normalization during quantification. RNA was transferred to Hybond N+ membranes (Amersham) in 1× TBE using the Mini Trans-Blot Cell apparatus (Bio-Rad) according to the manufacturer's instructions. Blots were prehybridized in Ultrahyb (Ambion) prior to addition of probe. RNA probes were transcribed from PCR-derived templates with T7 promoters using ^32^P-UTP and T7 polymerase (Promega) according to the manufacturer's instructions. Ambion Decade ladder labeled with ^32^P-ATP was run alongside RNA samples to provide estimations for the sizes of RNA bands. Hybridzation was carried out at 65°C overnight followed by washing 3× with low stringency buffer (2× SSC+0.05% SDS) wash at room temp, followed by washing 3× with high stringency buffer (0.2×SSC+0.05% SDS) at 65°C. Blots were then exposed to phosphor storage screens (Fuji) overnight. The image was subsequently read on a FLA-9000IR scanner. When reporting quantification, measurements taken from the phosphor screen after exposure were divided by fluorescent measurements of the 5S rRNA taken prior to transfer to normalize signal for loading using the MultiGage software (Fuji).

### qRT-PCR

Total RNA was purified from cultures grown under the indicated conditions using the mirVana RNA purification kit. Total RNA was treated with DNAase with the TURBO-DNAfree kit (Ambion) prior to reverse transcription. cDNA used as template was generated using iScript complete kit (BioRad) from 2 µg of total RNA using random hexamers. Quantitative PCR was run using Strategene Mv3005P equipment and MxPro qPCR software. Each sample was measured in technical triplicate. In all cases, controls lacking reverse transcriptase were included to assess DNA contamination, all results were either below the baseline of detection, or were subtracted from values obtained with those templates.

### Western blots

For western blot analysis of TcpF and GFP expression, strains carrying the TcpF C-terminal FLAG allele or the TarB-GFP fusion were grown under the indicated conditions at which times 2 mL culture aliquots were removed. Culture aliquots were immediately centrifuged at 10,000× g for 5 minutes to pellet cells, and supernatants were removed. Cell pellets were boiled in 50 µL (static timepoints and plasmid induction experiments) or 100 µL (4 h aeration timepoint) of SDS loading buffer (50 mM Tris-HCl, pH 6.8, 2% SDS, 0.5% bromophenol blue, 10% glycerol, 100 mM βME). Samples were cooled and a volume adjusted for differences in OD was loaded on an SDS-polyacrylamide gel electrophoresis (PAGE) gel and run 90 minutes at 125 V. Proteins were transferred to a nitrocellulose membrane at 25 V for 1 h. Membranes were loaded onto the SNAP-ID Western blotting system (Millipore) and blocked with 1× NAP blocking agent (G Biosciences) diluted in PBS+0.01% Tween-20. Primary antibody to the FLAG peptide (Invitrogen) or against GFP (Abcam) was added to the membrane 1∶600 or 1∶1200 respectively, diluted in 3 mL 1× NAP block for 10 minutes and the membrane was washed with 90 mL PBS+0.01% Tween-20. Secondary antibody (Invitrogen) (Cy5 conjugated goat anti mouse for anti-FLAG blots or Cy5 conjugated goat anti rabbit for GFP blots,) was added to the membrane at 1∶600 and diluted in 3 mL 1× NAP block for 10 minutes and the membrane was washed with 90 mL PBS+0.01% Tween-20. Bands were visualized using the Cy5 setting the FLA-9000IR. After visualization of TcpF-FLAG, blots were stripped by incubating in 20 mL acid stripping buffer (25 mM glycine pH 2, 1% SDS) shaking for 30 minutes followed by washing 2× with 20 mL PBS+0.01% Tween-20. After stripping, blots were reprobed as above with primary anti-OmpU at 1∶600 in 1× NAP block and secondary Cy5 conjugated goat anti-rabbit (Invitrogen) again in 1× NAP block and scanned on Cy5 setting on the FLA-9000IR.

Fluorescence measurements were quantified using MultiGage software (Fuji). Measurements TcpF-FLAG bands, adjusted for area and background, were divided by fluorescence measurements of corresponding OmpU bands adjusted for area and background. Loading-adjusted fluorescence values were then standardized to wild type expression and reported as fold expression of TcpF relative to wild type expression. The experiment shown is representative of six biological replicates.

### Mouse infections

Single strain infections and competition assays in infant mice, LB broth and filter sterilized pond water were performed with the TarB unmarked deletion strain (AC3744) (LacZ^+^) and wild type with a *lacZ* deletion (AC3745) for 24 h as described [57]. Inputs for competition assays and single strain infections were prepared by growth overnight on LB plates containing the appropriate antibiotics followed by resuspension in LB to an approximate density of 1×10^3^/µL as measured by OD, mixing of equal volumes of either culture (for competition experiments) then inoculation of infant mice by oral gavage. Samples from pond water incubations were prepared as described above, mixed in equal volumes and then used for innocualtion of infant mice. Immediately after inoculation, input ratios and total CFU were determined by plating on LB plates containing 5-bromo-4-chloro-3-indolyl-D-galactopyranoside (X-gal). The target input dose for all experiments was 10^5^ bacteria/mouse, although over the course of the experiments doses ranged between 10^4^ and 10^6^. Results are shown by the competition index (CI), which is the ratio of mutant CFU to wild type CFU normalized for the input ratio. To show complementation *in trans* in all assays in this study, Δ*tarB* derivatives (LacZ^+^) were complemented with either p*tarB* or ptarB* and were competed against the respective isogenic strain (LacZ^−^) carrying the pMMB67EH plasmid alone. CIs for these experiments are expressed as the ratio of mutant to complemented CFU corrected for input. To assess plasmid loss frequency, output plates were replica plated onto LB agar plates containing streptomycin and ampicillin at 100 µg/mL and X-Gal at 40 µg/mL to determine plasmid containing CFUs, and LB agar plates containing streptomycin and X-Gal to determine total CFUs.

### Growth curves

Growth of strains was determined by measured OD using a Bio-Tek microplate reader. Cultures grown overnight in LB plus streptomycin and (ampicillin at 50 µg/mL for complemented strains) or M9 glucose plus streptomycin and (ampicillin at 50 µg/mL for complemented strains) were resuspended to an OD of 0.01 in the respective media and pipetted into a 96-well plate in triplicate. Each growth curve was performed in biological triplicate. Bacteria were grown with aeration for 17 h at 37°C in the microplate reader with the OD being read every 17 minutes.

## Supporting Information

Figure S1 ***In vitro*** **analysis of the Δ** ***tarB*** **mutant and complemented strains.** **A**) *In vitro* competitions in LB between the Δ*tarB* and parental strains show no difference in fitness. In addition, the Δ*tarB* strain complemented with p*tarB* or containing empty vector show no significant difference during growth in LB. The Δ*tarB* strain was also competed against wild type for 24 h in pond water and again the Δ*tarB* strain showed no significant difference in fitness (one sample t-test). **B**) Shown is the median value of growth curves performed in biological triplicate with each individual sample being analyzed in technical triplicate. In either LB or in M9 minimal medium with glucose, the Δ*tarB* mutant showed no difference in growth rate when compared to the parental strain. We also measured the growth rate of complemented strains (Δ*tarB* [pMMB] and Δ*tarB* [p*tarB*]) in both LB and M9 minimal media and these also show no changes in growth rate.
(TIF)

Figure S2 **The RNA chaperone Hfq likely plays no role in TarB stability or in its interaction with TcpF transcript.** A) The TarB promoter-GFP fusion was made in strains deleted for *toxT* and carrying arabinose inducible ToxT on a plasmid in both the Hfq^+^ and Hfq^−^ backgrounds. These strains were then used to measure expression from the *tarB* promoter-*gfp* fusion, TarB from an intact native allele, expression of *toxT* from the plasmid, and expression of *tcpF*, which is the target of TarB by qRT-PCR. Data reported is the relative expression of those transcripts, adjusted for *rpoB* in the Hfq^+^ strain relative to the Hfq^−^ strain. Although expression of all transcripts were higher at 20 minutes post induction in the Hfq^+^ strain, there were similar before induction and after 40 minutes of ToxT induction. Adjusted for *toxT* expression, no differences were observed between Hfq^+^ and Hfq^−^ strains for expression of *tcpF* and *gfp* from the *tarB* promoter-*gfp* fusion. B) A northern blot for TarB was carried out on the same RNA samples used in Panel A for qRT-PCR, the results indicate that there is no large difference in steady state levels of the TarB sRNA in the Hfq^+^ and Hfq^−^ strains, suggesting that Hfq has no role in stabilizing the sRNA. C) Results from Panel A were confirmed by western blot for GFP in samples taken from the same experiment. The results indicate that, adjusted for loading, the two strains are expressing similar amounts of GFP from the *tarB*-*gfp* fusion prior to induction and at 40 minutes, indicating the *tarB*-*gfp* fusion is activated by expression of ToxT, as expected.
(TIF)

Figure S3 **TarB expression under anaerobic conditions.** A) Expression of *tcpF*, *toxT*, *cadC* and *gfp* from the *tarB*-*gfp* fusion. The *toxT* and anaerobically upegulated *cadC* genes were followed by qRT-PCR over the course of AKI induction. Shown are median expression values of technical triplicates, adjusted for the *rpoB* loading control relative to the two hour time point of AKI induction. Results indicate that *toxT* and *tcpF* have reached near maximal induction at 3 hours of static growth and expression of the *tarB*-*gfp* fusion and *cadC* show the most dramatic increases between 3 and 4 hours. This result was confirmed at the protein level by western blot of the wildtype strain carrying the TcpF-FLAG fusion taken through the same AKI induction experiment: loading was adjusted for OD of the culture instead of OmpU protein because levels of OmpU change with activation of ToxR. B) Both wildtype and Δ*toxT* strains were grown in buffered media containing glucose either in 2 mL culture tubes with aeration (+O_2_) or 10 mL sealed culture tubes prepared in an anaerobic chamber (−O_2_) at 37°C to early stationary phase. RNA was extracted and blotted for TarB. The results indicate that TarB is upregulated under anaerobic growth conditions independent of *toxT* when adjusting for loading.
(TIF)

Figure S4 **Quantitation of TcpF-FLAG by western blot.** Western blotting to quantitate TcpF-FLAG was performed a total of six times for each strain (including the shown example). For each experiment, the TcpF-FLAG fluorescence was divided by OmpU fluorescence and each experimental sample was normalized to the wildtype for that experiment by being set equal to one. Normalized fluorescence values were log transformed and evaluated by one sample T-test against one, the normalized wildtype value. In this analysis only the Δ*tarB* (p*tarB*) strains and ΔtarBtcpF* (ptarB*) had mean normalized fluorescence values significantly different from one.
(TIF)

Figure S5 **Expression of TcpF and TarB during pond water incubations.** A) Strains carrying the C-terminal TcpF-FLAG translational fusion or *tarB*-*gfp* (ASV) transcriptional fusion were incubated in pond water for the indicated amounts of time then lysed by boiling in SDS-loading buffer. Samples were then blotted with anti-FLAG and anti-GFP antibodies, loading was adjusted for OD as OmpU levels and were difficult to detect with our anti-OmpU antibody during pond water incubation. Levels of TcpF protein appear to decline over the course of pond water incubation, this effect was absent in the Δ*tarB* mutant. B) Expression from the TarB promoter as measured by GFP protein expression from the TarB-GFP fusion as measured by western blot, however, does not vary greatly over the course of pond water incubation.
(TIF)

Table S1 **Oligonucleotides.** Sequences of all oligonucleotides used as primers for PCR in this study.
(DOCX)

Table S2 **Plasmids.** Information about all plasmids utilized in this study and their origin.
(DOCX)

Table S3 **Strains.** The genotypes of all strains of *V. cholerae* utilized or constructed for this study.
(DOCX)

Table S4 **Linkers used for high throughput sequencing.** Sequences of linkers used to ligate sRNAs or gDNA for the construction of Illumina libraries.
(DOCX)

Table S5 **sRNA cluster location, abundance and length.** For each sRNA cluster, the location aligned to the N16961 genome (reference NC_002505 for chromosome 1, NC_002506 for chromosome 2) is given along with the relative abundance (see Materials and Methods for calculation), minimum and maximum predicted length of the putative sRNA.
(XLS)

Table S6 **Sites of enrichment in determined by ToxT genome wide binding assay.** Sites calculated to be enriched (see Materials and Methods for calculation) in Illumina libraries used in the ToxT pulldown assay versus the libraries sequenced prior to pulldown. The locations of the enriched sites are given aligned to the N16961 genome (reference NC_002505 for chromosome 1, NC_002506 for chromosome 2). The length of the site, maximum enrichment over background, the position of maximum enrichment, average coverage and expected coverage of each site is given in the adjacent columns. Values for Poisson distributions, Z scores and which technical replicate each site was found in, are given in the next columns. Finally, sites that overlap between the libraries are marked, and overlapping sites within libraries are scored to determine the approximate total enriched sites.
(XLS)
