# Supplementary figures and images for: A Genome-Wide Approach to Discovery of Small RNAs Involved in Regulation of Virulence in Vibrio cholerae
Source: PLoS Pathog. 2011 Jul 14;7(7):e1002126. doi: 10.1371/journal.ppat.1002126 (PMC3136459; doi:10.1371/journal.ppat.1002126)

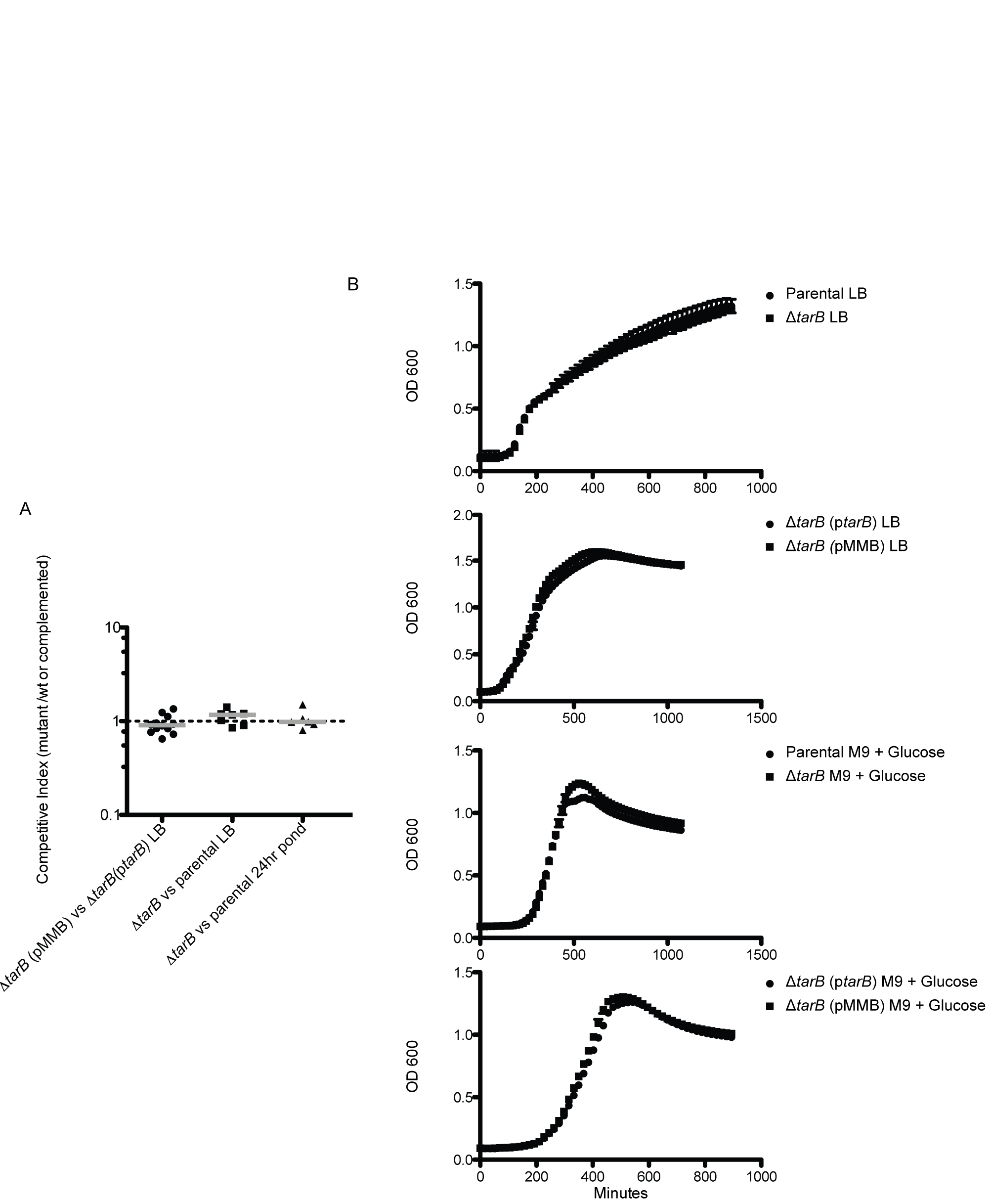

Supplement: Figure S1 — In vitro analysis of the Δ tarB mutant and complemented strains. A) In vitro competitions in LB between the ΔtarB and parental strains show no difference in fitness. In addition, the ΔtarB strain complemented with ptarB or containing empty vector show no significant difference during growth in LB. The ΔtarB strain was also competed against wild type for 24 h in pond water and again the ΔtarB strain showed no significant difference in fitness (one sample t-test). B) Shown is the median value of growth curves performed in biological triplicate with each individual sample being analyzed in technical triplicate. In either LB or in M9 minimal medium with glucose, the ΔtarB mutant showed no difference in growth rate when compared to the parental strain. We also measured the growth rate of complemented strains (ΔtarB [pMMB] and ΔtarB [ptarB]) in both LB and M9 minimal media and these also show no changes in growth rate. (TIF) [file ppat.1002126.s001.tif]

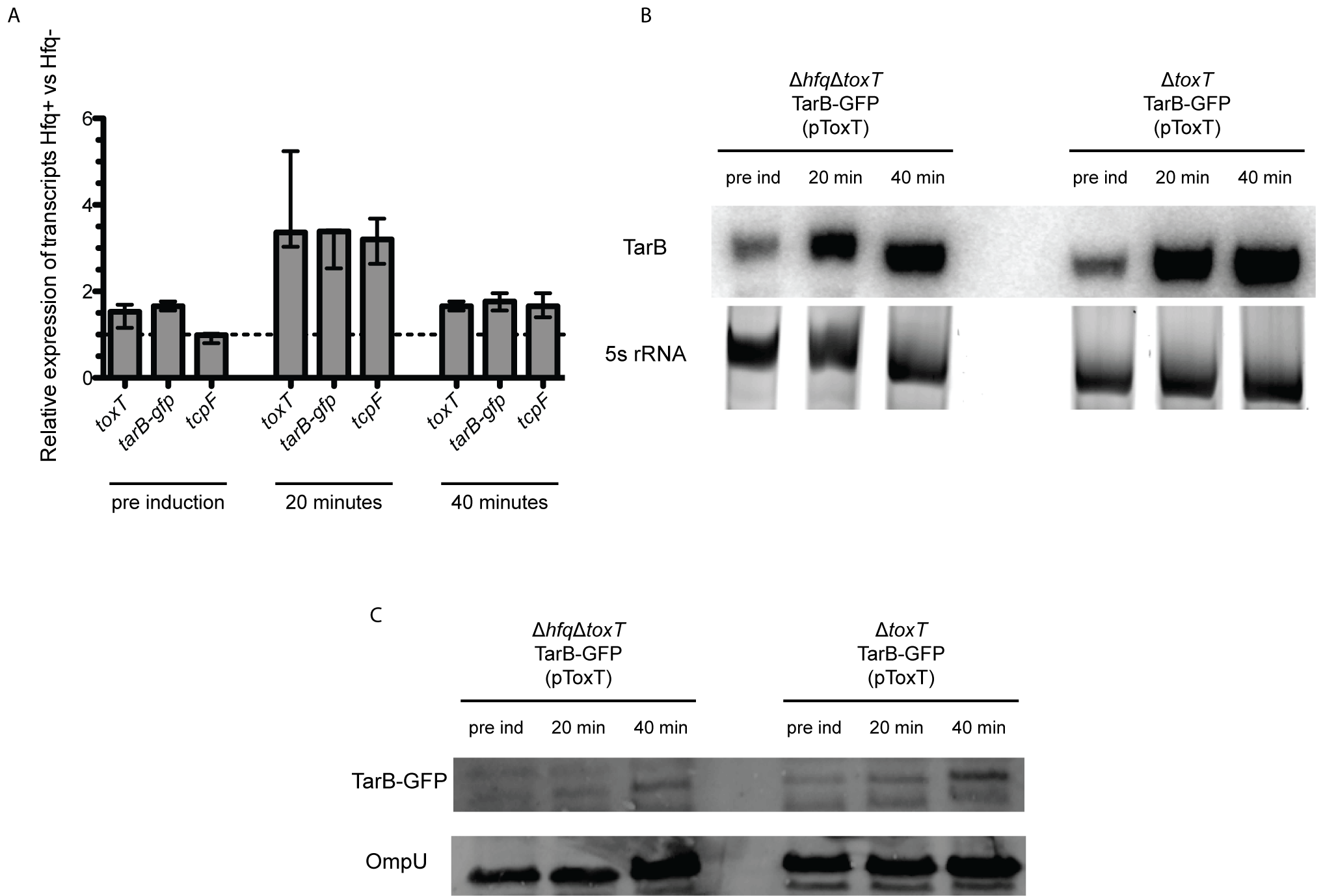

Supplement: Figure S2 — The RNA chaperone Hfq likely plays no role in TarB stability or in its interaction with TcpF transcript. A) The TarB promoter-GFP fusion was made in strains deleted for toxT and carrying arabinose inducible ToxT on a plasmid in both the Hfq+ and Hfq− backgrounds. These strains were then used to measure expression from the tarB promoter-gfp fusion, TarB from an intact native allele, expression of toxT from the plasmid, and expression of tcpF, which is the target of TarB by qRT-PCR. Data reported is the relative expression of those transcripts, adjusted for rpoB in the Hfq+ strain relative to the Hfq− strain. Although expression of all transcripts were higher at 20 minutes post induction in the Hfq+ strain, there were similar before induction and after 40 minutes of ToxT induction. Adjusted for toxT expression, no differences were observed between Hfq+ and Hfq− strains for expression of tcpF and gfp from the tarB promoter-gfp fusion. B) A northern blot for TarB was carried out on the same RNA samples used in Panel A for qRT-PCR, the results indicate that there is no large difference in steady state levels of the TarB sRNA in the Hfq+ and Hfq− strains, suggesting that Hfq has no role in stabilizing the sRNA. C) Results from Panel A were confirmed by western blot for GFP in samples taken from the same experiment. The results indicate that, adjusted for loading, the two strains are expressing similar amounts of GFP from the tarB-gfp fusion prior to induction and at 40 minutes, indicating the tarB-gfp fusion is activated by expression of ToxT, as expected. (TIF) [file ppat.1002126.s002.tif]

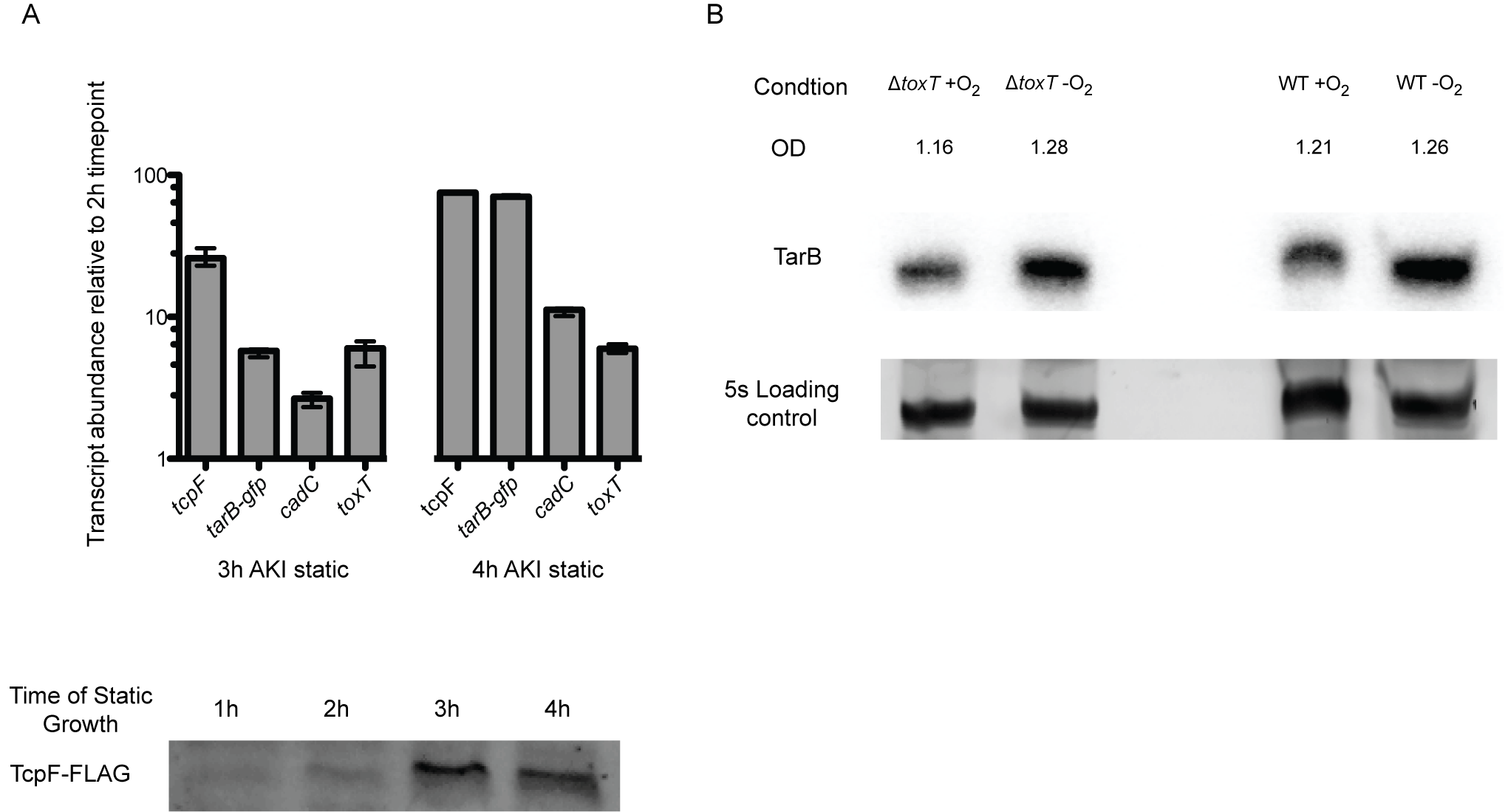

Supplement: Figure S3 — TarB expression under anaerobic conditions. A) Expression of tcpF, toxT, cadC and gfp from the tarB-gfp fusion. The toxT and anaerobically upegulated cadC genes were followed by qRT-PCR over the course of AKI induction. Shown are median expression values of technical triplicates, adjusted for the rpoB loading control relative to the two hour time point of AKI induction. Results indicate that toxT and tcpF have reached near maximal induction at 3 hours of static growth and expression of the tarB-gfp fusion and cadC show the most dramatic increases between 3 and 4 hours. This result was confirmed at the protein level by western blot of the wildtype strain carrying the TcpF-FLAG fusion taken through the same AKI induction experiment: loading was adjusted for OD of the culture instead of OmpU protein because levels of OmpU change with activation of ToxR. B) Both wildtype and ΔtoxT strains were grown in buffered media containing glucose either in 2 mL culture tubes with aeration (+O2) or 10 mL sealed culture tubes prepared in an anaerobic chamber (−O2) at 37°C to early stationary phase. RNA was extracted and blotted for TarB. The results indicate that TarB is upregulated under anaerobic growth conditions independent of toxT when adjusting for loading. (TIF) [file ppat.1002126.s003.tif]

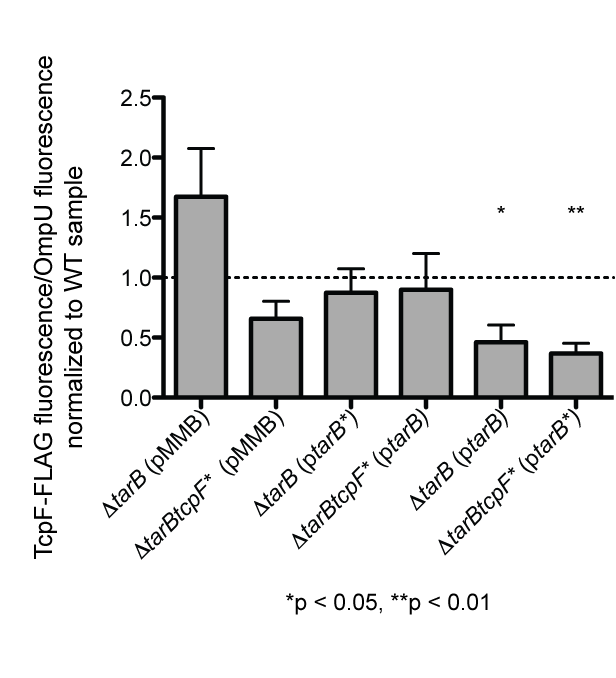

Supplement: Figure S4 — Quantitation of TcpF-FLAG by western blot. Western blotting to quantitate TcpF-FLAG was performed a total of six times for each strain (including the shown example). For each experiment, the TcpF-FLAG fluorescence was divided by OmpU fluorescence and each experimental sample was normalized to the wildtype for that experiment by being set equal to one. Normalized fluorescence values were log transformed and evaluated by one sample T-test against one, the normalized wildtype value. In this analysis only the ΔtarB (ptarB) strains and ΔtarBtcpF* (ptarB*) had mean normalized fluorescence values significantly different from one. (TIF) [file ppat.1002126.s004.tif]

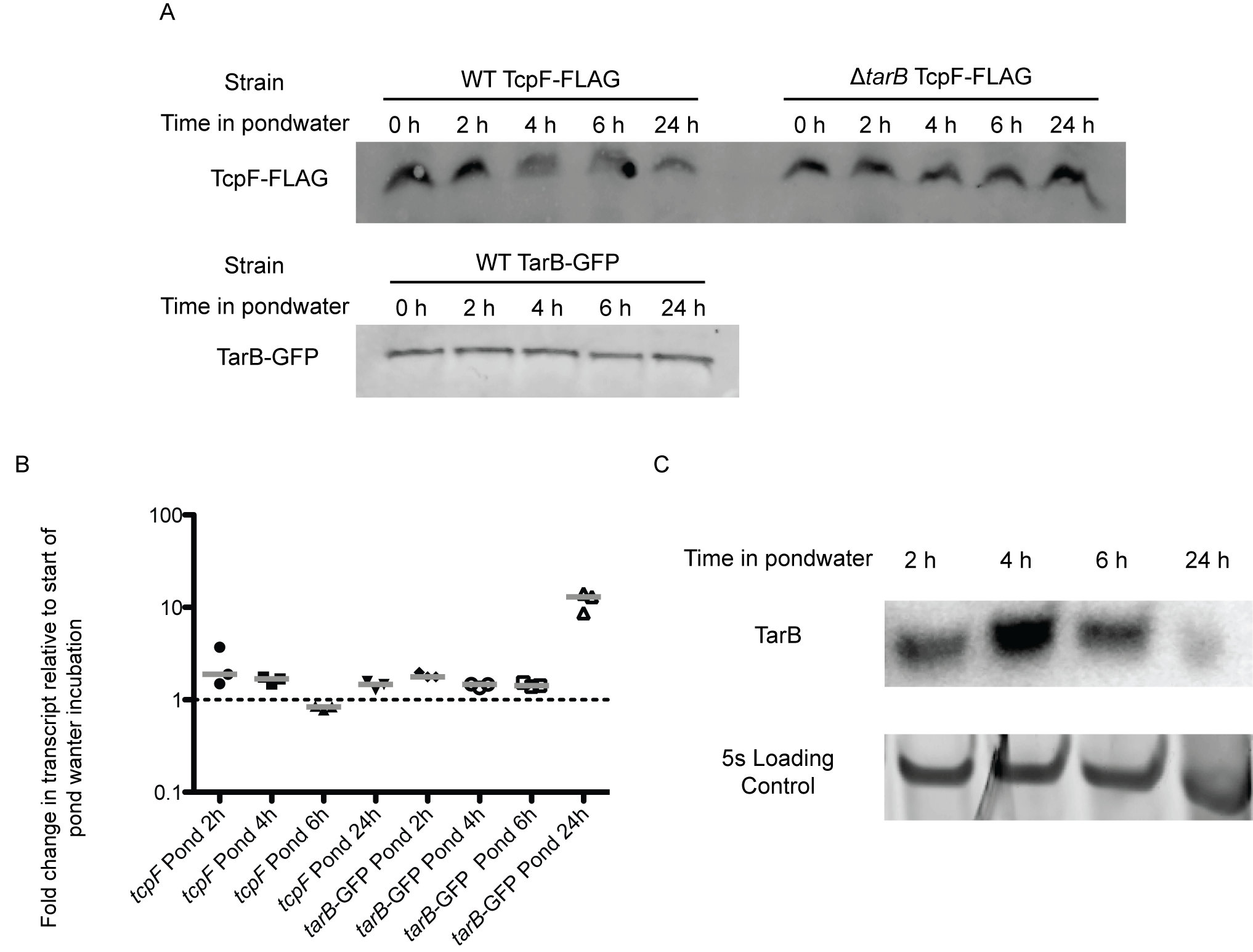

Supplement: Figure S5 — Expression of TcpF and TarB during pond water incubations. A) Strains carrying the C-terminal TcpF-FLAG translational fusion or tarB-gfp (ASV) transcriptional fusion were incubated in pond water for the indicated amounts of time then lysed by boiling in SDS-loading buffer. Samples were then blotted with anti-FLAG and anti-GFP antibodies, loading was adjusted for OD as OmpU levels and were difficult to detect with our anti-OmpU antibody during pond water incubation. Levels of TcpF protein appear to decline over the course of pond water incubation, this effect was absent in the ΔtarB mutant. B) Expression from the TarB promoter as measured by GFP protein expression from the TarB-GFP fusion as measured by western blot, however, does not vary greatly over the course of pond water incubation. (TIF) [file ppat.1002126.s005.tif]
