# Supplementary material for: A Genome-Wide Approach to Discovery of Small RNAs Involved in Regulation of Virulence in Vibrio cholerae
Source: PLoS Pathog. 2011 Jul 14;7(7):e1002126. doi: 10.1371/journal.ppat.1002126 (PMC3136459; doi:10.1371/journal.ppat.1002126)
Supplement: Table S1 — Oligonucleotides. Sequences of all oligonucleotides used as primers for PCR in this study. (DOCX) [file ppat.1002126.s006.docx]

Table S1. Primers

| Name | Sequence |
| --- | --- |
| ToxT_NcoI_F | GCCCATGGTATCTTCAGAGTAGAACGCAATGA |
| ToxT_XbaI_R | GCTCTAGATTATTTTTCTGCAACTCCTGTCA |
| IL1a | CTCTTTCCCTACACGACGCTCTTCCGATCTGATTGATGGTGCCTACAG |
| IL1b | GGCATTCCTGCTGAACCGCTCTTCCGATCTGTCCTTGGTGCCCGAGTG |
| IL1c | GGCATTCCTGCTGAACCGCTCTTCCGATCTGCTGGAATTCGCGGTTAAA |
| IL2a | AATGATACGGCGACCACCGAGATCTACACTCTTTCCCTACACGACGCTCTTCCGATCT |
| IL2b | CAAGCAGAAGACGGCATACGAGATCGGTCTCGGCATTCCTGCTGAACCGCTCTTCCGATCT |
| ΔtoxT_F1_(XbaI) | GCTCTAGATTCTCTGCTCGGCTTTTAGC |
| ΔtoxT_R1 | GTAAACGTATTCCATTACATTGCGTTCTACTCTGAAGATATATA |
| ΔtoxT_F2 | CAGAGTAGAACGCAATGTAATGGAATACGTTTACTTGATCCTA |
| ΔtoxT_R2_(XbaI) | GCTCTAGATTTGACACATCGACCTTGGA |
| ΔtoxT_F0 | CTACGGATTCAAGGGGGAG |
| ΔtoxT_R0 | TCCTGAACGTCATCTAGTGGT |
| toxT_NdeI_F | GCCATATGATTGGGAAAAAATCTTTTCAAACTAA |
| toxT_BamHI_R | GCGGATCCTTATTTTTCTGCAACTCCTGTCAACAT |
| MtlS_Rev | CCGTTGGTGATTCCATTCG |
| MtlS_For | TCCCCCGTTGGATGTTCCG |
| ΔtarA_F1-SphI | GCGCATGCTCGCTTGTATGTTTGGACGA |
| ΔtarAR1 | TTCGGTTTAGCACTCCCTAACTTTATTTTCCTAAAGACAAA |
| ΔtarA_F2 | GGGAGTGCTAAACCGAATGAATTATAATGAGAATTACTTT |
| ΔtarA_R2-SphI | GCGCATGCCCCCCAAGCTTTTAATTTTT |
| T7_tarB_F | TAATACGACTCACTATAGGGCGCCAAAAAGTGCTTAATCG |
| tarB_R | AAAACAAAATCATCTTTCATAACAGC |
| ΔtarB_F1_XbaI | GCTCTAGAGTGTTGGTGCTGCACACTCT |
| ΔtarB_R1 | AGCAATGTAACCAACCTCAAATATTAACCCTTAGGATATTC |
| ΔtarB_F2 | TTGAGGTTGGTTACATTGCTTTTTAACGCTCTTGTTTCTATTTAAGC |
| ΔtarB_R2_XbaI | GCTCTAGACCTTTCCCAAATTGAGTTCG |
| Δhfq_F1_xbaI | GCTCTAGAACTGATTTATCGAGGGATGG |
| Δhfq_R1 | GATCCAGAAATGGGTCTTGTAGAGATTGCC |
| Δhfq_F2 | CCCATTTCTGGATCGTCCAGCAGAGAAGTCT |
| Δhfq_R2_xbaI | GCTCTAGATTACGCAAAGTAGGATCGAG |
| T7_tarA_F | TAATACGACTCACTATAGGG CCAAACGTAAGGGGCAAAAT |
| tarA_R | ATAATTCATTCGGTTTAGCACTCC |
| tarA_comp_F | GATGTGAAAAATCAGCTTTTATCGT |
| tarA_comp_R_ | ATTTGCAATCTAATTCTGCAGTTG |
| xbaI_tarB_ comp_F | GCTCTAGATTGAGGTTGGTTACATTGCTATAA |
| sacI_tarB _comp_R | GCGAGCTCGCTTAAATAGAAACAAGAGCGTTAAAA |
| TarB_promoter_F | TGTATGTTTATAGTGCCAGTAT |
| TarB_promoter_R_-100 | CATAAGCTTAAATAGAAACAAGA |
| TarB_promoter_R_-300 | TTTAAAGATAGAGTGATCGCG |
| 4.5s_F | CTGGTCCTCCCGCAACAC |
| 4.5s_R | GAGACCCCAGCCACATC |
| TcpF_mut_F1_-_xbaI | GCTCTAGAGAGGGAGTGGGCATCTATGA |
| tcpF_mut_F2 | TTCTAGTTTATAGTGAGGTATTATGAGATATAAAAAAACCTTAATG |
| tcpF_mut_R1 | ATACCTCACTATAAACTAGAACTTAGTTTATCAACGAGCG |
| tcpF_mut_R2_-xbaI | GCTCTAGACCGTTAAGTTGCCACTAGGC |
| tcpF_mut_F0 | TGAAAATTATCTCCAAGAAGTATAGGC |
| tcpF_mut_R0 | TTGACCACTTGTAACCATTATGC |
| tarB_mut_R1 | CATGATATGTTACAAGCTGACCTATAAGCACTTTTTGGCGCACTGCGG |
| tarB_mut_F2 | TTATAGGTCAGCTTGTAACATATCATGAGGTAACCGCTCATGTATG |
| tcpF_qt_F | TGGTGCAATGATCGCAGTAT |
| tcpF_qt_R | CCGTTAAGTTGCCACTAGGC |
| Vca_0638_qt_F | CGGTTTAGTGCGCCATTATT |
| Vca_0638_qt_R | CCATACACTTCCGCCAGAAT |
| Vc0177_qt_F | TAACGGTGAAGGGAGTGGTC |
| Vc0177_qt_R | TGGTTCCAGTTCAGGGAATC |
| Vc0937_qt_F | TTGGTTGATGTGCAAGGTGT |
| Vc0937_qt_R | TCAGCGACTTTCAAATCACG |
| Vc2506_qt_F | CAGCCAAGCTCAACAAAACA |
| Vc2506_qt_R | CATCAAACAGGCTCAAAGCA |
| Vca0686_qt_F | CCTACTCTAGCTTCTGTATGCTGG |
| Vca0686_qt_R | GAATGATGCTGATCAGTGAGCC |
| CadC_qt_F | TATGTGGTGACGGTGCCTAA |
| CadC_qt_R | TTCGGCTTGCTTGATTTCTT |
| tcpF_C-FLAG-_R | TTAGCTCTAGTTATTTGTCATCGTCATCCTTGTAGTCTTTAAAGTTCTCTGAATATGC |
| Olj139 | AATGATACGGCGACCACCGAGATCTACACTCTTTCCCTACACGA |
| Olj140 | CAAGCAGAAGACGGCATACGAGATCGGTCTCGGCATTCCTGCTGAAC |
| Fgfp2 | GCGAGCTCTTTAGGATTTATTAAAATGCGTAAAGGAGAAGAACTT |
| RGFP2 | GCGCCCGGGCTAAACTGATGCAGCGTAGTTTTCGTCGTTTGCTGCAGGCCTTTTGTATAGTTCATCCATGCC |
