# Supplementary material for: A Genome-Wide Approach to Discovery of Small RNAs Involved in Regulation of Virulence in Vibrio cholerae
Source: PLoS Pathog. 2011 Jul 14;7(7):e1002126. doi: 10.1371/journal.ppat.1002126 (PMC3136459; doi:10.1371/journal.ppat.1002126)
Supplement: Table S2 — Plasmids. Information about all plasmids utilized in this study and their origin. (DOCX) [file ppat.1002126.s007.docx]

Table S2. Plasmids

| Name | Description | Reference |
| --- | --- | --- |
| pToxT | pBAD24 backbone with toxT ORF and RBS cloned into the NcoI/XbaI site | This study |
| pToxT (ΔHLH) | pBAD24 back bone with toxT ΔHLH ORF and RBS loned into the NcoI/XbaI site | This study |
| pMMB 67EH | Apr, IncQ broad-host-range cloning vector | [[1](#_ENREF_1)] |
| p*tarB* | pMMB67EH with fragment containing the tarB sequence, promoter and terminator cloned into the SacI/XbaI sites | This study |
| p*tarB** | pMMB67EH with fragment containing the tarB sequence, promoter and terminator cloned into the SacI/XbaI sites | This study |
| pMAL-TEV-His-thr-*toxT* | pMAL vector from NEB modified to contain a TEV protease cleavage site, a 6x his tag and a thrombin cleavage site in between MBP and toxT, the toxT ORF was cloned into the NdeI/BamHI site of this vector | This study |
| p*tarA* | Topo vector pCR 2.1 containing the tarA sequence including promoter and previously described toxboxes | This study |
| pGEM-T | Empty vector containing the colE1 origin, used as no insert control for competitions against ptarA containing strain | Promega |
| pGP704 cm tcpF C-FLAG | Derivitive of the pGP704 plasmid containing the CAT gene swapped for bla and the C-terminal 300 base pairs of the tcpF ORF with in-frame FLAG tag cloned into the EcoRV/KpnI sites. Used for generating the C-Terminal FLAG fusion to TcpF | This study |
| ptarB-300 | Topo vector pCR2.1 containing the +3 to -380bp of the tarB promoter | This study |
| pTarB-GFP(ASV) | Derivitive of the pGP704 plasmid containing the CAT gene swapped for bla and the -380 to +3bp of the TarB promoter cloned ahead of the Gfp(ASV) [[2](#_ENREF_2),[3](#_ENREF_3)]allele in the SmaI site | This study |

1. Morales VM, Backman A, Bagdasarian M (1991) A series of wide-host-range low-copy-number vectors that allow direct screening for recombinants. Gene 97: 39-47.

2. Andersen JB, Sternberg C, Poulsen LK, Bjorn SP, Givskov M, et al. (1998) New unstable variants of green fluorescent protein for studies of transient gene expression in bacteria. Appl Environ Microbiol 64: 2240-2246.

3. Nielsen AT, Dolganov NA, Rasmussen T, Otto G, Miller MC, et al. (2010) A bistable switch and anatomical site control Vibrio cholerae virulence gene expression in the intestine. PLoS Pathog 6.
