# Supplementary material for: A Genome-Wide Approach to Discovery of Small RNAs Involved in Regulation of Virulence in Vibrio cholerae
Source: PLoS Pathog. 2011 Jul 14;7(7):e1002126. doi: 10.1371/journal.ppat.1002126 (PMC3136459; doi:10.1371/journal.ppat.1002126)
Supplement: Table S3 — Strains. The genotypes of all strains of V. cholerae utilized or constructed for this study. (DOCX) [file ppat.1002126.s008.docx]

Table S3. Strains

| Name | Genotype | Source |
| --- | --- | --- |
| AC53 | Wiltype E7946 El Tor, O1 Ogawa | Laboratory Collection |
| AC3745 | Δ*lacZ* | This study |
| AC3763 | Δ*toxT* | This study |
| AC3744 | Δ*tarB* | This study |
| AC3748 | Δ*tarB*Δ*lacZ* | This study |
| AC3746 | Δ*tarA* | This study |
| AC3749 | Δ*tarA*Δ*lacZ* | This study |
| AC3757 | Δ*tarB*Δ*toxT* | This study |
| AC3794 | Δ*tarBtcpF** | This study |
| AC3795 | Δ*tarB*Δ*lacZtcpF** | This study |
| AC3765 | Δ*hfq* | This study |
| AC468 | Δ*toxR* (ΔHLH) C6709 El Tor O1 Inaba | Laboratory Collection |
| AC522 | Δ*tcpPH*::*KanR* res-tet-res C6709 El Tor O1 Inaba | Laboratory Collection |
| AC3780 | *tarB** | This study |
| AC3781 | *tcpF** | This study |
| AC3782 | *tarB*tcpF** | This study |

All strains, except where noted, are derivatives of wildtype E7946
