# Supplementary material for: A Genome-Wide Approach to Discovery of Small RNAs Involved in Regulation of Virulence in Vibrio cholerae
Source: PLoS Pathog. 2011 Jul 14;7(7):e1002126. doi: 10.1371/journal.ppat.1002126 (PMC3136459; doi:10.1371/journal.ppat.1002126)
Supplement: Table S4 — Linkers used for high throughput sequencing. Sequences of linkers used to ligate sRNAs or gDNA for the construction of Illumina libraries. (DOCX) [file ppat.1002126.s009.docx]

Table 4. Barcoded adapters and sRNA cloning linkers for constructing Illumina libraries

| Name | Sequence | barcode |
| --- | --- | --- |
| Linker1 | rAppCTGTAGGCACCATCAAT/3ddC/ |  |
| Linker2 | rAppCACTCGGGCACCAAGGA/3ddC/ |  |
| Linker3 | rAppTTTAACCGCGAATTCCAG/3ddC/ |  |
| BC1a | AATGATACGGCGACCACCGAGATCTACACTCTTTCCCTACACGACGCTCTTCCGATCTAACCT | AACC |
| BC1b | P-GGTTAGATCGGAAGAGCGGTTCAGCAGGAATGCCGAGACCGATCTCGTATGCCGTCTTCTGCTTG | AACC |
| BC2a | AATGATACGGCGACCACCGAGATCTACACTCTTTCCCTACACGACGCTCTTCCGATCTTTGGT | TTGG |
| BC2b | P-CCAAAGATCGGAAGAGCGGTTCAGCAGGAATGCCGAGACCGATCTCGTATGCCGTCTTCTGCTTG | TTGG |
| BC3a | AATGATACGGCGACCACCGAGATCTACACTCTTTCCCTACACGACGCTCTTCCGATCTCCAAT | CCAA |
| BC3b | P-TTGGAGATCGGAAGAGCGGTTCAGCAGGAATGCCGAGACCGATCTCGTATGCCGTCTTCTGCTTG | CCAA |
